# Fluorescent detection of hydrogen sulfide (H_2_S) through the formation of pyrene excimers enhances H_2_S quantification in biochemical systems

**DOI:** 10.1016/j.jbc.2022.102402

**Published:** 2022-08-19

**Authors:** Manuela Pose, Kearsley M. Dillon, Ana Denicola, Beatriz Alvarez, John B. Matson, Matías N. Möller, Ernesto Cuevasanta

**Affiliations:** 1 Laboratorio de Enzimología, Instituto de Química Biológica, Facultad de Ciencias, Universidad de la República, Montevideo, Uruguay; 2 Laboratorio de Fisicoquímica Biológica, Instituto de Química Biológica, Facultad de Ciencias, Universidad de la República, Montevideo, Uruguay; 3 Centro de Investigaciones Biomédicas (CEINBIO), Universidad de la República, Montevideo, Uruguay; 4 Department of Chemistry and Macromolecules Innovation Institute, Virginia Tech, Blacksburg, Virginia, USA; 5 Unidad de Bioquímica Analítica, Centro de Investigaciones Nucleares, Facultad de Ciencias, Universidad de la República, Montevideo, Uruguay

**Keywords:** fluorescence, fluorescent probes, hydrogen sulfide, H_2_S, pyrene excimers, quantification, Cys, cysteine, DHLA, dihydrolipoic acid, DNS-Az, 5-(dimethylamino)naphthalene-1-sulfonyl azide, DTT, DL-dithiothreitol, GSH, glutathione, H_2_S, hydrogen sulfide, MEPB, 2-(maleimido)ethyl 4-pyrenylbutanoate, TCEP, tris(2-carboxyethyl)phosphine, Tris–ACN, Tris–acetonitrile

## Abstract

Hydrogen sulfide (H_2_S) is produced endogenously by several enzymatic pathways and modulates physiological functions in mammals. Quantification of H_2_S in biochemical systems remains challenging because of the presence of interferents with similar reactivity, particularly thiols. Herein, we present a new quantification method based on the formation of pyrene excimers in solution. We synthesized the probe 2-(maleimido)ethyl 4-pyrenylbutanoate (MEPB) and determined that MEPB reacted with H_2_S in a two-step reaction to yield the thioether-linked dimer (MEPB)_2_S, which formed excimers upon excitation, with a broad peak of fluorescence emission centered at 480 nm. In contrast, we found that the products formed with thiols showed peaks at 378 and 398 nm. The difference in emission between the products prevented the interference. Furthermore, we showed that the excimer fluorescence signal yielded a linear response to H_2_S, with a limit of detection of 54 nM in a fluorometer. Our quantification method with MEPB was successfully applied to follow the reaction of H_2_S with glutathione disulfide and to quantify the production of H_2_S from cysteine by *Escherichia coli*. In conclusion, this method represents an addition to the toolkit of biochemists to quantify H_2_S specifically and sensitively in biochemical systems.

Hydrogen sulfide (H_2_S)^a^ has been associated to the origin and the evolution of life (1, 2, 3). It occurs naturally in volcanoes, natural gas, and sulfur springs and is generated by bacterial decomposition of biological material, as observed in sewer systems and swamps. One of the first properties known was its high toxicity, recognized for centuries and causative of numerous diseases in occupational settings (4, 5). Exposure of humans to mild atmospheric levels of H_2_S (>10 ppm) is harmful. Different industrial processes handle high amounts of H_2_S, representing a hazard for humans in case of accidents (5, 6). Despite its toxicity, H_2_S was explored as a therapeutic agent (7). Later on, it was found to be produced endogenously in mammals with effects on the nervous and vascular systems (8, 9, 10, 11). Efficient and rapid enzymatic pathways for H_2_S formation and consumption have been identified in mammals, reinforcing the concept that H_2_S is biologically relevant (12, 13, 14). These routes must be strictly regulated since high levels of H_2_S inhibit mitochondrial respiration (15). The mechanisms underlying H_2_S signaling are currently under scrutiny, with metal centers and oxidized thiol derivatives likely participating in the initial sensing of H_2_S (16, 17). In this regard, the development of chemical tools to deliver and detect H_2_S and other reactive sulfur species is warranted to better explore their biochemistry (18, 19, 20).

The detection and quantification of H_2_S in biological systems are challenging. Several estimations of the steady-state level of H_2_S in tissues have been done and corrected as interferences were identified (21). Among other confounding factors, the volatility of H_2_S and the presence of other sulfur compounds in the samples (thiols, bound forms of sulfide, and partially oxidized sulfur species) complicate the attainment of accurate results. Some reliable determinations inform basal values of 6 to 80 nM H_2_S in most murine tissues (22). Therefore, prime challenges in H_2_S detection are the sensitivity and selectivity of the method.

Although there is a wide variety of quantification methods, several of them show low selectivity in biochemical systems. The available methodologies take advantage of some features of H_2_S: (1) it is a gas under normal conditions, (2) it is a reducing agent, (3) it forms insoluble salts with metal ions, and (4) it has *bis*-nucleophilic character, that is, it is both a nucleophile and a nucleophile precursor: the nucleophilic reaction of H_2_S with a suitable electrophile forms a thiol, which is a new nucleophile that can react itself with another electrophile. Some of these features are common to other compounds also present in biochemical samples, so methods are at risk of lacking specificity. One crucial concern is the interference by thiols, present in millimolar levels in biochemical samples. Thiols share some chemical properties with H_2_S, particularly their nucleophilicity and oxidizability.

An early and not very sensitive (but useful) method involved the formation of dark precipitates with lead cations on soaked paper sheets, which enabled to test the presence of H_2_S in the atmosphere. This approach is still being used to sense H_2_S gas in cell cultures or reaction mixtures but shows poor linearity and sensitivity (23). The methylene blue method is the gold standard for environmental measurements (24). Samples are incubated with *N*,*N*-dimethyl-*p*-phenylenediamine and ferric ions in a strongly acidic medium to synthesize methylene blue. It is a convenient and sensitive method, but, in biochemical samples, the extreme conditions used could modify equilibria or release bound forms of sulfide, such as iron–sulfur clusters (25). Also, the presence of high concentrations of thiols or other reductants in the sample interferes with the yield of this reaction (26). A more sensitive method is gas chromatography coupled to a chemiluminescence sulfur detection system (21), which provides reliable and sensitive results, but specialized equipment is required and samples need to be withdrawn from the headspace. Electrochemical devices have also been developed (27). H_2_S-sensitive electrodes allow continuous monitoring in solution with good sensitivity. Their selectivity toward H_2_S relies on the permeability of a silicone polymer membrane.

Fluorescent probes are promising tools because of potentially high sensitivity. Some reported probes use azide or nitro derivatives of rhodamine or dansyl, which are able to form fluorescent amines upon reduction by H_2_S (28, 29). Unfortunately, thiols can also potentially reduce these probes and thus interfere with the detection of H_2_S. Other fluorescent probes are attached to a chelator with Cu^2+^ or Zn^2+^ as a quencher (30, 31). If H_2_S is present, it pulls the cations out, enabling the fluorescence emission. Despite the high sensitivity of these probes, the selectivity to H_2_S with respect to thiols is based on the relative stability of the metal–chelator complex. A third strategy takes advantage of the nucleophilicity of H_2_S. Usually, haloalkanes on a fluorescent scaffold are used as electrophiles, for example, monobromobimane. Since other nucleophiles (thiols, thiosulfate, and sulfite) are also able to react with the probe, the fluorescent products are separated and quantified by chromatography (32). Last, an interesting approach takes advantage of the *bis*-nucleophilic character of H_2_S, avoiding the interference of thiols and improving the selectivity. H_2_S is able to react with a first electrophilic center present in a fluorogenic scaffold forming a thiol, which can react with a second electrophilic group producing a fluorescent moiety (33, 34).

Herein, a novel fluorescence method is proposed based on the reaction between H_2_S and an *N*-ethylmaleimide-linked pyrene derivative (2-(maleimido)ethyl 4-pyrenylbutanoate [MEPB]) under mild conditions. Pyrenes are high-quantum-yield fluorophores used to label diverse molecules (*e.g.*, maleimide derivatives used to detect thiols (35, 36, 37)). Conveniently, the attached maleimide acts as an intramolecular quencher that is deactivated after reaction with nucleophiles. The ability to form excimers constitutes a remarkable property of pyrenes (38, 39). Excimers occur when an electronically excited pyrene forms a complex with a ground-state pyrene and results in an emission at a higher wavelength than that of an unassociated pyrene molecule (monomer). The formation of excimers requires spatial proximity to allow the π-stacking of two pyrenes. We hypothesized that given the *bis*-nucleophilic nature of H_2_S, it could react with two molecules of MEPB, bring the pyrenes closer, and induce excimer emission (Equations 1 and 2).

(1) $${\text{H}}_{2}\text{S}\;+\;\text{MEPB}\;\to \;\text{MEPB-SH}$$

(2) $$\text{MEPB-SH}\;+\;\text{MEPB}\;\to \;{\left(\text{MEPB}\right)}_{2}\text{S}$$

Thiols, as nucleophiles, would also react with the MEPB probe to cancel the self-quenching of fluorescence induced by the maleimide group. However, H_2_S is able to react with two molecules of MEPB, anchoring each other covalently and favoring the formation of excimers upon excitation. Excimer emission is red shifted over 100 nm with respect to the emission of the product with thiols (35, 38, 40). This large Stokes shift would permit sensitive and single-step quantification of H_2_S even in the presence of thiols.

## Results

### Synthesis of MEPB

**Figure 1:**
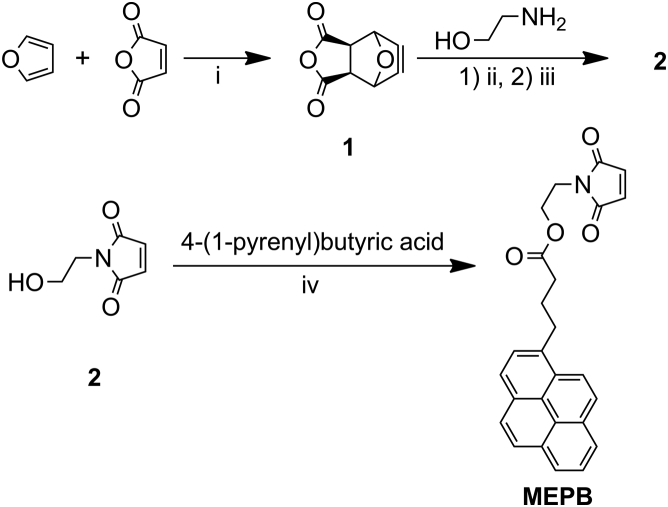
**Synthetic route to MEPB.** Reaction conditions: (i) diethyl ether, 12 h, room temperature (RT); (ii) methanol, 16 h, reflux; (iii) toluene, 8 h, 90 °C; (iv) 1-ethyl-3-carbodiimide, 4-dimethylaminopyridine, tetrahydrofuran, 16 h, RT. MEPB, 2-(maleimido)ethyl 4-pyrenylbutanoate.

Preliminary experiments using *N*-(1-pyrene)maleimide showed that the dithiol DTT could successfully form excimers. However, H_2_S did not form excimers (Fig. S1), likely because of steric hindrance, indicating the need for a longer linker. To achieve a flexible pyrene-based profluorophore while retaining the ability to react with H_2_S and form excimers, we synthesized a profluorophore with a four-carbon spacer in between the pyrene and maleimide groups, linked *via* an ester. The synthesis of this new probe, termed MEPB, was accomplished in three steps through the use of a Diels–Alder/imide formation/retro-Diels–Alder sequence to avoid side products formed because of conjugate addition of an amine to maleic anhydride (Fig. 1). First, furan was treated with maleic anhydride in a Diels–Alder reaction to form an oxanorbornene anhydride (Fig. S2). Next, this anhydride was condensed with ethanolamine and then heated to induce a retro-Diels–Alder reaction, regenerating furan along with the desired *N*-(2-hydroxyethyl)maleimide product (Fig. S3). Finally, *N*-(2-hydroxyethyl)maleimide was combined with 4-(1-pyrenyl)butyric acid using an 1-ethyl-3-(3-dimethylaminopropyl)carbodiimide coupling reaction in the presence of 4-dimethylaminopyridine as catalyst, affording MEPB (Figs. S4–S6).

### Formation of excimers after reaction of MEPB with H_2_S

**Figure 2:**
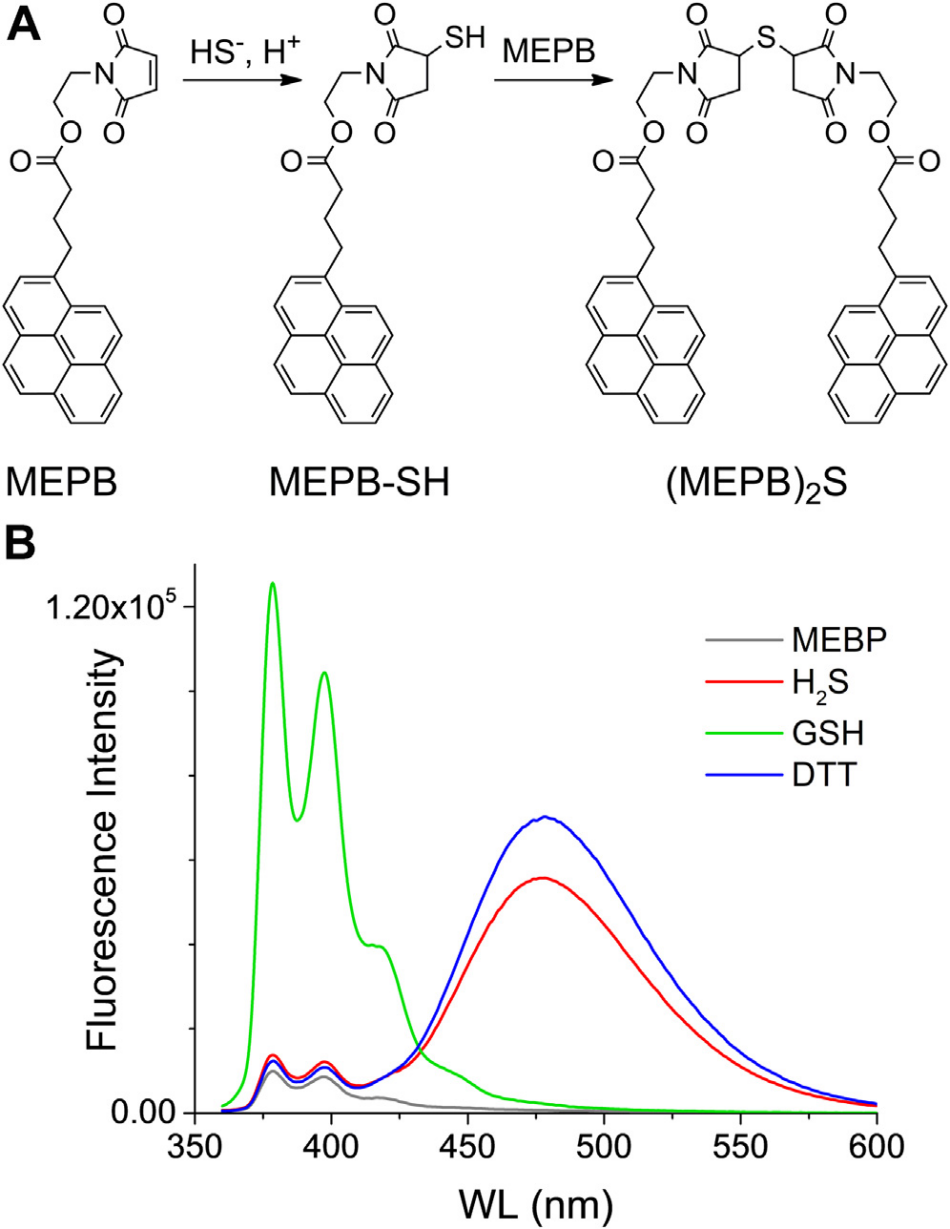
**Excimer formation with pyrene derivatives.***A*, MEPB reacts sequentially with H_2_S to yield first a thiol intermediate (MEPB-SH) and then a thioether-bridged dimer ((MEPB)_2_S); the long linker provides flexibility to the molecule and permits the formation of excimers. *B*, emission spectra of MEPB derivatives (λ_ex_ = 345 nm, corrected, with polarizers as described in Measurement of H2S with MEPB section). MEPB (25 μM) was mixed with H_2_S, GSH, or DTT (12.5, 25, and 12.5 μM, respectively) in Tris–ACN, for 20 min at room temperature, and diluted 50-fold before measurement in the ISS instrument. H_2_S, hydrogen sulfide; MEPB, 2-(maleimido)ethyl 4-pyrenylbutanoate; Tris–ACN, Tris buffer (0.1 M, pH 8.5) and acetonitrile (1:1 volume mixture).

MEPB showed a low intrinsic fluorescence because of intramolecular quenching by the maleimide. The quenching was canceled upon reaction with GSH, DTT, and H_2_S (Fig. 2). The product of GSH was a thioether that showed an emission spectrum characteristic of pyrene, with two main peaks at 378 and 398 nm. In contrast, the product of the dithiol DTT showed a broad peak at 480 nm, indicating the formation of pyrene excimers. Notably, H_2_S reacted with two molecules of MEPB, yielding a thioether-bridged MEPB dimer ((MEPB)_2_S) that was able to form excimers, confirming our working hypothesis (Fig. 2). The emission spectrum of this product was very similar to that of DTT, with a broad peak at 480 nm. The formation of excimers was further confirmed by the fact that both excitation and absorption spectra were the same for all species (38). For instance, the excitation spectra obtained with emission at 480 nm of DTT and H_2_S derivatives were identical to the excitation spectra of the monomer obtained by emission at 378 nm of the GSH derivative (Fig. S7). Also, the absorption spectra of the products were indistinguishable from the spectrum of the original probe (Fig. S7) (41). The products obtained after the incubation of MEPB with H_2_S were sceparated by reversed-phase HPLC and a peak with a higher retention time than MEPB with emission at 480 nm (λ_ex_ = 345 nm) was observed, consistent with the formation of (MEPB)_2_S (Fig. S8). Furthermore, high-resolution mass spectrometry confirmed the formation of this product (Fig. S9). The fluorescence emission was sensitive to the solvent composition, and the greatest emission of excimers from (MEPB)_2_S was obtained using the 1:1 volume mixture of Tris buffer (0.1 M, pH 8.5) and acetonitrile (Tris–ACN, Fig. S10).

### Kinetics of the reaction between H_2_S and MEPB

**Figure 3:**
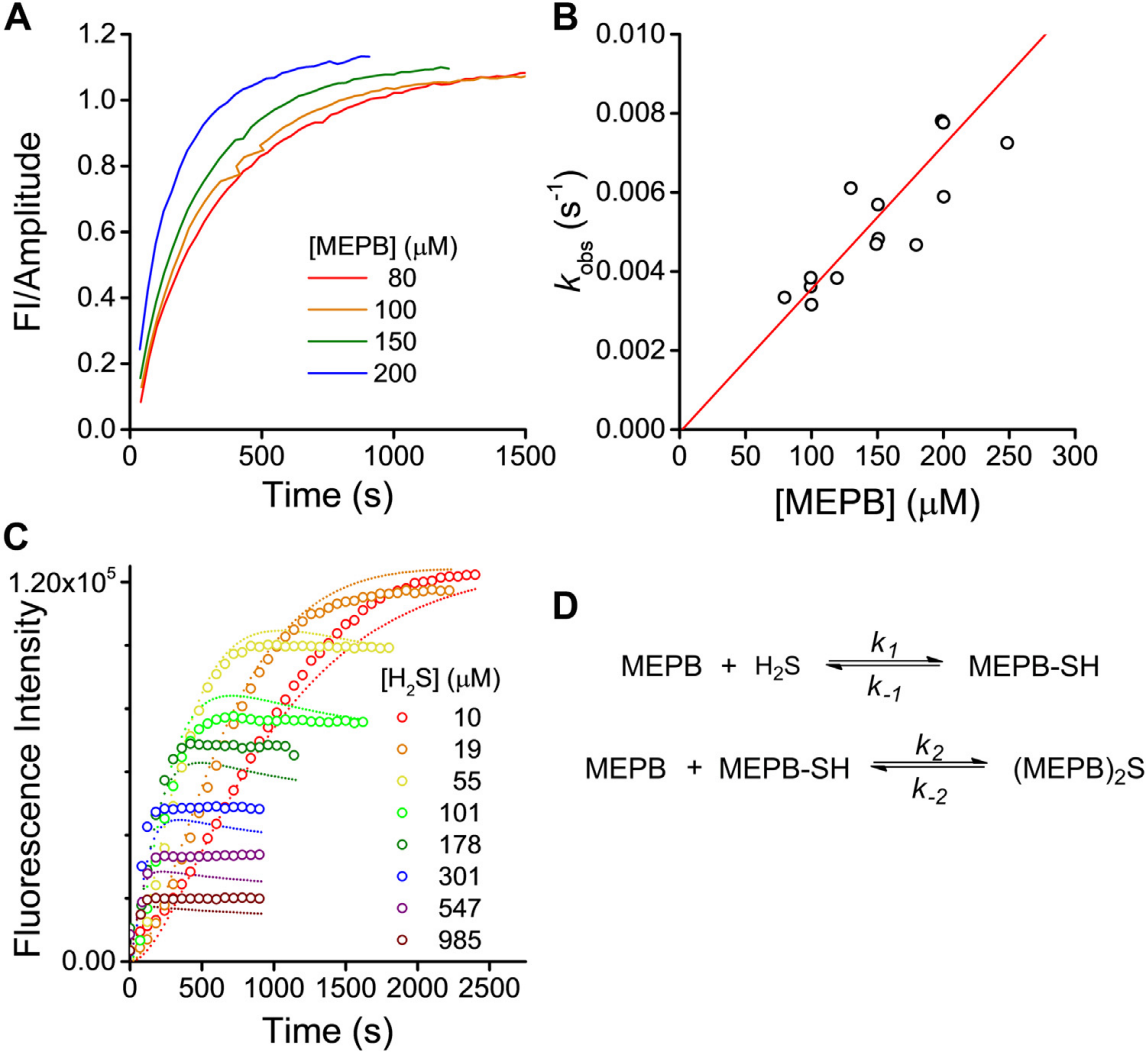
**Kinetics of the reaction of MEPB and H**_**2**_**S.***A*, time courses of the reaction between H_2_S (5 μM) and MEPB in excess (80–250 μM) in Tris–ACN at 25 °C (λ_ex_ = 345 nm, λ_em_ = 480 nm), obtained in cuvettes with a path length of 4 mm. Exponential rate constants (*k*_obs_) were obtained from exponential plus straight line functions fitted to the data. *B*, exponential rate constants (*k*_obs_) of excimer formation *versus* concentration of MEPB. The second-order rate constant was 36 ± 5 M^−1^ s^−1^ (pH 8.5, 25 °C). *C*, representative time courses of excimer formation when MEPB (500 nM) was mixed with variable concentrations of H_2_S in excess (10–985 μM) in Tris–ACN at 25 °C (λ_ex_ = 345 nm, λ_em_ = 480 nm). *Dottedlines* represent the best fit to a model consisting of two reversible reactions using DynaFit, which yielded rate constants of *k*_1_ = 40 ± 1 M^−1^ s^−1^, *k*_−1_ = (4.0 ± 0.9) × 10^−4^ s^−1^, *k*_2_ = (5.1 ± 0.9) × 10^3^ M^−1^ s^−1^, and *k*_−2_ = (10 ± 2) × 10^−4^ s^−1^. *D*, proposed reaction scheme. H_2_S, hydrogen sulfide; MEPB, 2-(maleimido)ethyl 4-pyrenylbutanoate; Tris–ACN, Tris–acetonitrile.

To assess the time of incubation needed to complete the reaction between the H_2_S and MEPB, and to better understand the mechanism of the process, we performed kinetic studies. The formation of pyrene covalent dimers is not a one-step but a two-step process, so we determined the rate constants of both additions, of H_2_S to MEPB and of the thiol intermediate to MEPB (Fig. 2*A*). In a first approach, MEPB was used in a pseudo–first-order excess. The progress of the reaction was followed by changes in emission at 480 nm, which showed a single exponential behavior (Fig. 3*A*). The observed rate constants increased linearly with MEPB concentration (Fig. 3*B*) with a slope of 36 ± 5 M^−1^ s^−1^ at 25 °C (pH 8.5, 50% ACN). No lag time was observed in the time courses of excimer formation. This suggests that the rate constant for the second step (the reaction of the thiol intermediate with a second MEPB to form (MEPB)_2_S) is higher than the rate constant of the first step (the formation of MEPB–SH). Thus, the value of 36 ± 5 M^−1^ s^−1^ at 25 °C was assigned to the rate constant of the reaction of H_2_S with MEPB to form the thiol intermediate (*k*_*1*_).

If H_2_S is in large excess with respect to MEPB, a condition very unlikely to occur during H_2_S determinations, but useful to understand the mechanism of (MEPB)_2_S synthesis, the formation of the thiol intermediate is favored. In fact, the formation of monomers was evidenced by emission at 380 nm, which showed a monophasic profile (not shown). However, even under limiting concentrations of MEPB, excimers were also formed (Fig. 3*C*). This confirms that the second reaction has a relatively high rate constant. By lowering the concentration of H_2_S, kinetics became more complex, showing a biphasic behavior because of the change in the relative weight of the two reactions involved.

The addition of nucleophiles to maleimides is expected to be very favorable but potentially reversible. To interpret the reactions involved, a comparison between kinetic models was performed with the DynaFit software (BioKin, Ltd) (42) (see Supporting information). Fittings to kinetic traces allowed selecting a model based on the Akaike information criterion, consisting of two reversible reactions (Fig. 3*D*). The kinetic parameters obtained by fitting the data were *k*_1_ = 40 ± 1 M^−1^ s^−1^, *k*_−1_ = (4.0 ± 0.9) × 10^−4^ s^−1^, *k*_2_ = (5.1 ± 0.9) × 10^3^ M^−1^ s^−1^, and *k*_−2_ = (10 ± 2) × 10^−4^ s^−1^ (pH 8.5, 25 °C) (Fig. 3*C*). Of note, the value obtained for *k*_1_ was in good agreement with that obtained in Figure 3*A* and 3*B*. These results indicate that the rate constant of the first step of the reaction between H_2_S and MEPB to form the thiol MEPB-SH (*k*_*1*_) is 100 times lower than the subsequent reaction between MEPB-SH and a second molecule of MEPB (*k*_*2*_). This is expected from thiols being better nucleophiles than H_2_S (43). For comparison, a rate constant of 6.7 × 10^4^ M^−1^ s^−1^ was reported for the reaction of *N*-ethylmaleimide with β-mercaptoethanol (44). It can also be concluded that, although the reactions are reversible, the apparent equilibrium constants favor the formation of the adducts (10^5^ and 10^6^ M^−1^ for the first and second step, respectively). The low values of the reverse rate constants assure that the adducts remain stable during dilutions and measurements.

Control experiments were done to assess the stability of the solutions, since maleimides can undergo alkaline hydrolysis (45) and, in addition, MEPB contains an ester bond. Controls performed by incubating either MEPB or (MEPB)_2_S at different pHs indicated that incubations below pH 9 are safe from hydrolysis for up to 2 h (Figs. S11 and S8). Considering that the rate constant for the slow step of the reaction is 36 M^−1^ s^−1^, a concentration of MEPB of 200 μM in the detection solution would result in a reaction half-life of 96 s. Thus, 15 min of incubation represent nine half-lives, the reaction is 99.8% accomplished, and it can be assumed complete.

### Linearity of the response and limit of detection

The high absorption coefficient of pyrenes (ε_345_ = 40,000 M^−1^ cm^−1^) (46, 47) could represent a pitfall for quantification because of the inner filter effect. A high solution absorbance extinguishes the incident light lowering the excitation of the fluorophores. Thus, to check the upper limit of probe concentration to use during readings, serial dilutions of a solution containing (MEPB)_2_S and excess MEPB were measured (Fig. S12). While a pronounced inner filter effect was observed above 50 μM pyrene, a linear correlation was found below 8 μM (Fig. S12), thus setting an upper limit to the final concentration of MEPB recommended for measurements. Note that 8 μM would be the final concentration after dilutions; the MEPB concentration for the reactions with H_2_S can be higher.

**Figure 4:**
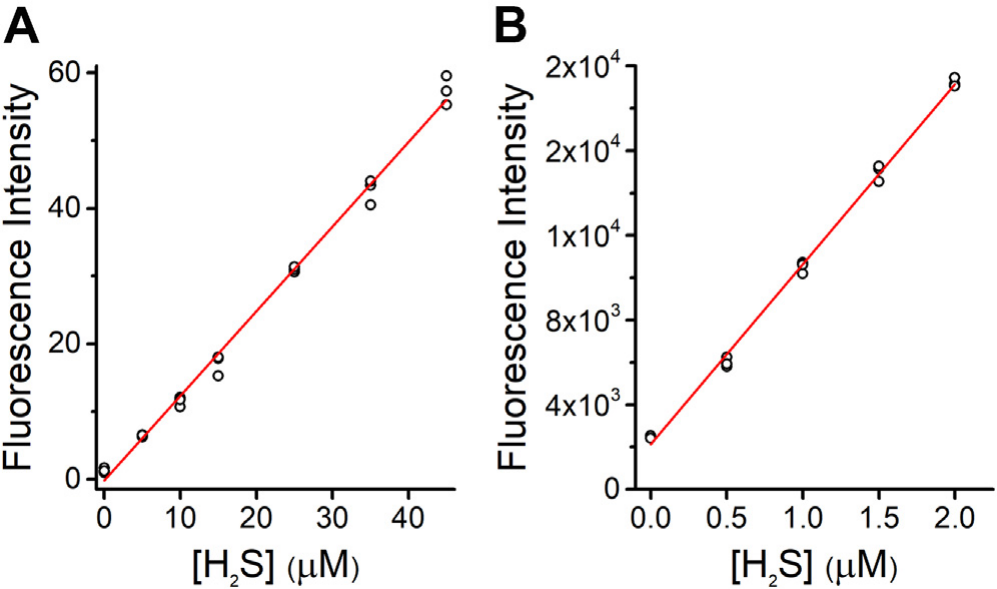
**Linearity of response.***A*, H_2_S was treated with MEPB according to the procedure stated in Measurement of H2S with MEPB section, and fluorescence intensity was measured in a plate reader. The detection and quantification limits estimated are 0.6 and 2.0 μM, respectively (*R*^2^ = 0.99396). *B*, low-range calibration curve following the same procedure as aforementioned, except that the measurements were done in an ISS fluorometer. In this case, the limits of detection and quantitation were 54 and 181 nM (*R*^2^ = 0.99716). Scatter plots were done in triplicates, and the best lines were obtained from linear regressions. The figures are representative examples of calibration curves performed dozens of times. H_2_S, hydrogen sulfide; MEPB, 2-(maleimido)ethyl 4-pyrenylbutanoate.

Calibration curves with known concentrations of H_2_S were performed to ascertain the linearity range and the sensitivity of the method (Fig. 4). The determinations of H_2_S were done with 200 μM MEPB to ensure complete reaction in a relatively short time, and measurements were done after diluting the sample 40 times (5 μM MEPB final concentration). A linear response was observed up to 20 μM H_2_S (initial concentration) in a plate reader (Varioskan) (Fig. 4*A*). Limits of detection and quantitation were estimated as 0.6 and 2.0 μM, respectively. Determination of submicromolar concentrations of H_2_S becomes noisier in the plate reader but could be better performed in a fluorometer (ISS) (Fig. 4*B*). In this instrument, the limit of detection and quantitation in the low range were estimated as 54 and 181 nM, respectively. The use of higher initial concentrations of probe (up to 400 μM) had no effect on the signal obtained from the same amount of the analyte.

### Specificity of the method

**Figure 5:**
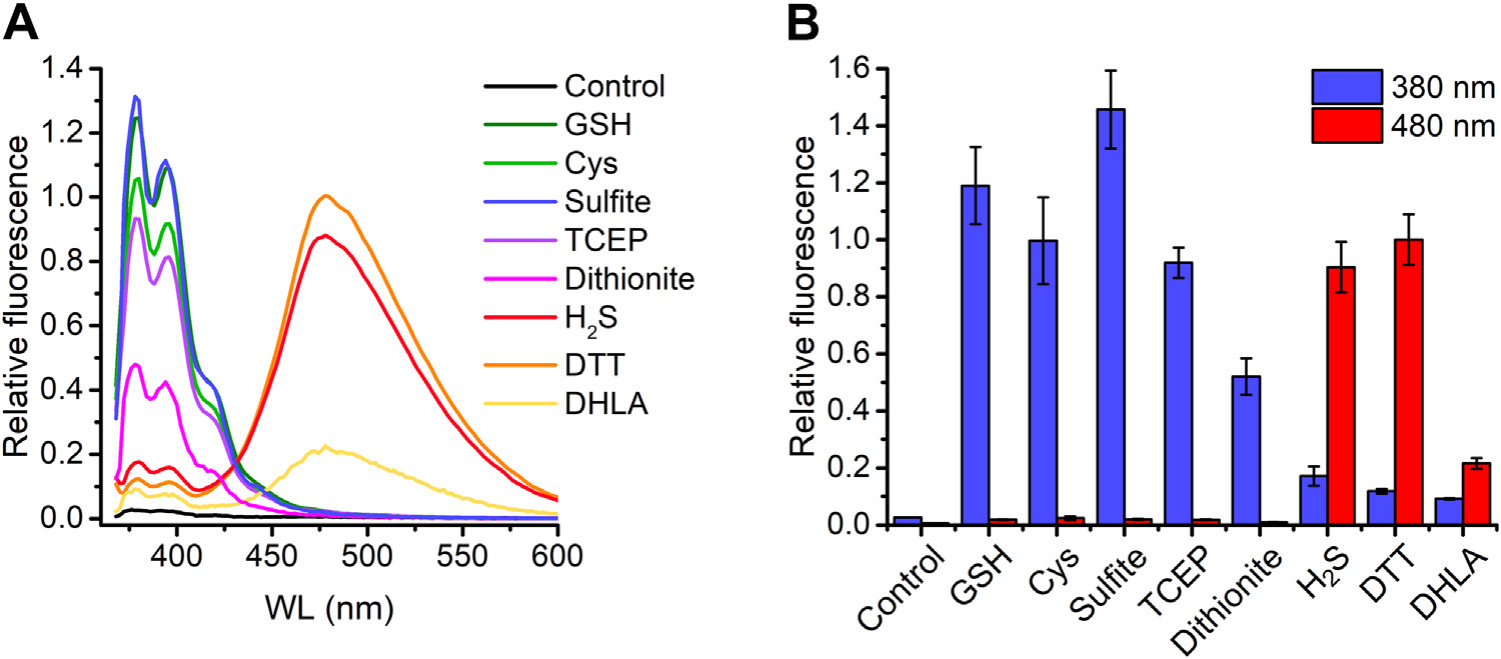
**Comparison of the emission spectra of MEPB with different reagents.***A*, emission spectra of the different reaction products between MEPB (200 μM) and GSH, cysteine, sulfite, TCEP, dithionite, H_2_S, DTT, or DHLA (50 μM each). The reaction was done following the procedure described in Measurement of H2S with MEPB section, and measurements were done in a plate reader (Varioskan). *B*, contribution of different nucleophiles to the emission at 380 and 480 nm. The experiment was done as in (*A*) (average ± standard deviation, n = 3). DHLA, dihydrolipoic acid; H_2_S, hydrogen sulfide; MEPB, 2-(maleimido)ethyl 4-pyrenylbutanoate; TCEP, tris(2-carboxyethyl)phosphine.

To assess the possible interference by other nucleophiles, we prepared mixtures with MEPB and evaluated the emission at 480 nm. As shown in Figure 5, *A* and *B*, monothiols like cysteine (Cys) and GSH, sulfite, and the reducing agents tris(2-carboxyethyl)phosphine (TCEP) and dithionite (which forms sulfite when oxidized by dioxygen), reacted with the probe but did not form excimers. Despite the high emission at 380 nm, the contribution at 480 nm was much lower than the signal produced by the product of H_2_S. The use of DTT, a reagent frequently added to biochemical systems to reduce thiols, must be avoided because of the interference observed due to excimer formation (monothiols, TCEP, or dithionite could be used instead). Dihydrolipoic acid (DHLA) may also contribute to excimer formation. However, in biological samples, DHLA is usually bound to proteins that will precipitate at the ACN concentration used to measure (MEPB)_2_S. Furthermore, the product of DHLA with MEPB also had low solubility in Tris–ACN, resulting in a lower than expected signal (Fig. 5*B*). Additional controls for detecting DHLA or other dithiols could include extensive purging with argon or nitrogen to eliminate the volatile H_2_S followed by determination of the possible presence of dithiols with MEPB.

The emission at 480 nm of the monomer resulting from 50 μM GSH addition is negligible, compared with the emission of low micromolar levels of the excimer resulting from H_2_S (Fig. S13). Although an increase in the concentration of thiols may cause an increase in the baseline at 480 nm, it is possible to measure the emission of the excimer at higher wavelengths, such as 520 nm, where the emission of monomers is even lower (Fig. S13).

**Figure 6:**
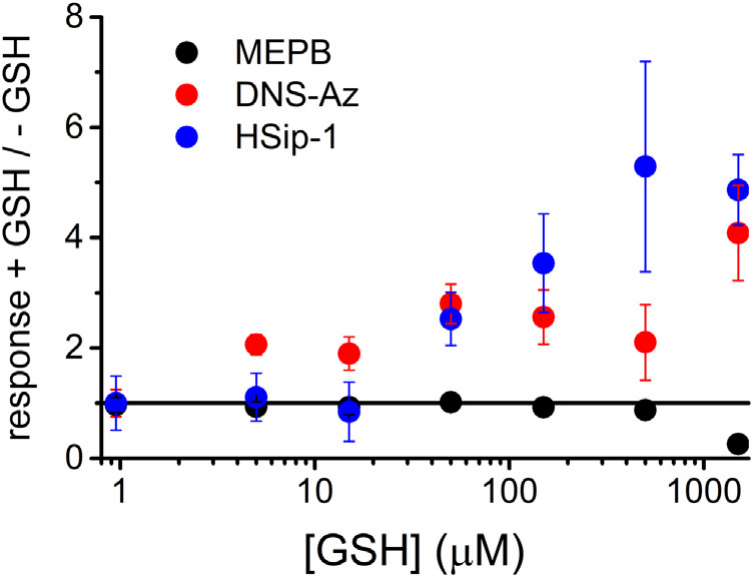
**Comparison of MEPB with other H**_**2**_**S-probes.** H_2_S (20 μM), in the presence of increasing concentrations of GSH (5–1500 μM), was submitted to three different treatments: MEPB method (n = 4), DNS-Az (n = 4), or HSip-1 (n = 3). Points represent the average ± standard deviation. DNS-Az, 5-(dimethylamino)naphthalene-1-sulfonyl azide; H_2_S, hydrogen sulfide; MEPB, 2-(maleimido)ethyl 4-pyrenylbutanoate.

With regard to the issue of thiol interference, the specificity of the MEPB method seems to be improved compared with alternative fluorescent probes (Fig. 6). Using 200 μM probe and 20 μM H_2_S in the presence of different concentrations of GSH, it was found that 5-(dimethylamino)naphthalene-1-sulfonyl azide (DNS-Az) was particularly sensitive to the presence of GSH, leading to an overestimation of H_2_S when assayed with more than 5 μM GSH. The profluorescent Cu(II)-complex HSip-1 (30) resulted in an overestimation of H_2_S when assayed with more than 50 μM GSH. Conversely, the presence of increasing concentrations of GSH produces minimal interference on the response of MEPB until the probe is the limiting reagent (Fig. 6). This is relevant not only for accurate quantification of H_2_S but also for avoiding false-positive responses.

### Using MEPB to monitor H_2_S in biochemical systems

**Figure 7:**
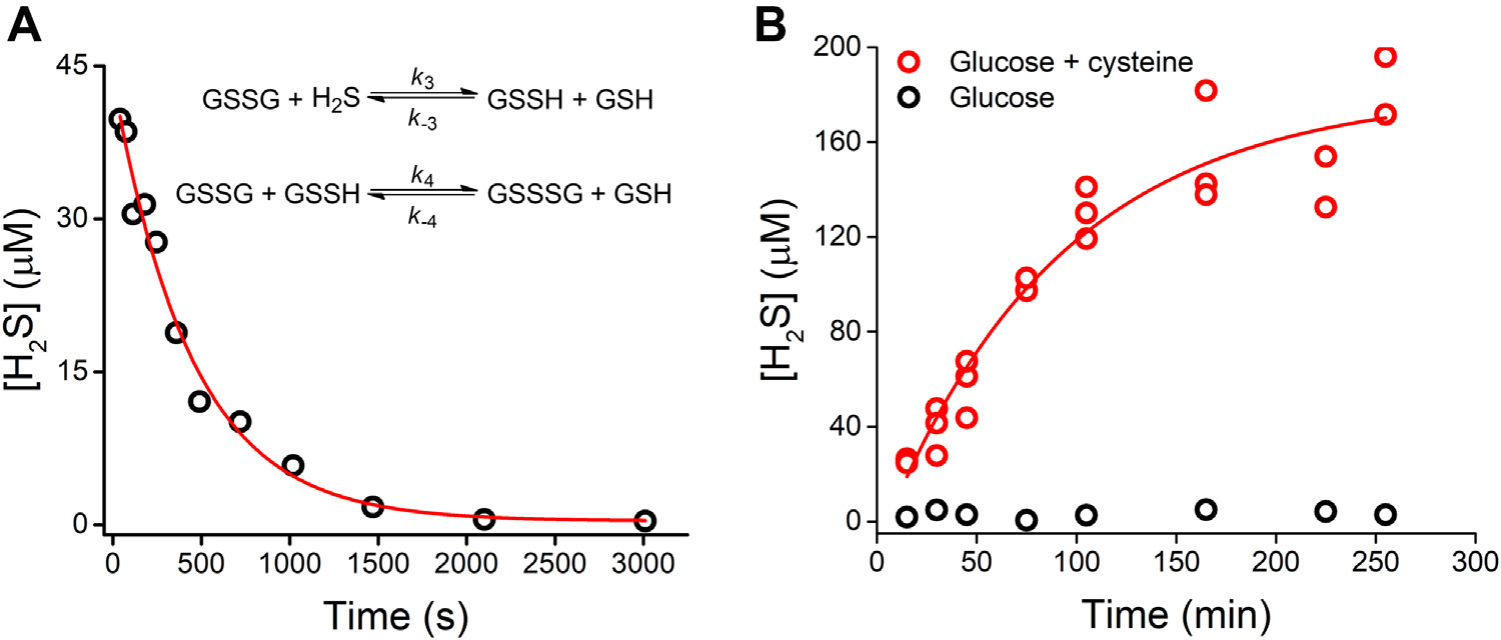
**Determination of H**_**2**_**S in biochemical systems.***A*, decay of H_2_S in the presence of GSSG. The time course of the reaction of H_2_S (54 μM) and GSSG (10 mM) in phosphate buffer (0.1 M, pH 7.4) at 25 °C was monitored by withdrawing aliquots along the incubation period and treating them according to Measurement of H2S with MEPB section. Representative course, n = 2. The reactions involved in H_2_S consumption are shown in the *inset*. *B*, formation of H_2_S by *Escherichia coli* cultures. *E. coli* suspensions (absorbance at 600 nm = 0.5) in bicine buffer (0.1 M, pH 8.0) were supplemented with glucose (2 g/l) and cysteine (200 μM) and incubated at 37 °C. At increasing times, cultures were centrifuged, aliquots were withdrawn from the supernatant, and analyzed according to Measurement of H2S with MEPB section (scatter plots obtained from three independent experiments and one control culture). H_2_S, hydrogen sulfide.

The reaction of H_2_S with GSSG is a potentially important reaction in biology leading to the formation of GSH and glutathione persulfide (GSSH), involved in H_2_S cell signaling transduction (43, 48). The disappearance of H_2_S in this reaction was monitored by MEPB detection (Fig. 7*A*). A single exponential equation was fitted to the decay of H_2_S, and a second-order rate constant of 0.20 ± 0.04 M^−1^ s^−1^ (pH 7.4, 25 °C) was obtained (*k*_3_), in very good agreement with previous reports (43, 48).

The prokaryote *Escherichia coli* expresses cysteine desulfhydrase and desulfurases, which could contribute to H_2_S formation from cysteine, but does not express sulfide quinone oxidoreductase or cysteine dioxygenase, which promote the oxidation of H_2_S and cysteine, respectively. MEPB allowed following the formation of H_2_S by the cells in the presence of cysteine. Using *E. coli* suspensions (absorbance 0.5 at 600 nm, ∼10^8^ cells/ml), 200 μM cysteine was consumed in 4 h and produced high yields of H_2_S (>90%) (Figs. 7*B* and S15). Therefore, MEPB can be used to measure H_2_S in processes of biochemical interest, even in the presence of thiols.

## Discussion

The use of an electrophile-linked pyrene derivative that takes advantage of the *bis*-nucleophilic reactivity of H_2_S to bring two pyrenes close enough to form excimers represents a novel strategy for H_2_S detection. The virtually null fluorescence of the probe and the large Stokes shift in the emission of the excimers in comparison to the monothiol-probe adduct makes this approach a convenient way to avoid or minimize interferences caused by thiols or other nucleophiles and reducing agents.

The described procedure clarifies and circumvents issues that are encountered with several methods using fluorescent probes but are usually not clearly exposed. Descriptions of novel methodologies usually focus on the response of probes to the analyte, but less effort is made to warn researchers about possible drawbacks. Here, the kinetics of the reactions, experimental conditions, and the potential interference by compounds with similar reactivity were carefully assessed.

In this method, a stepwise approach is used, where the H_2_S to be analyzed is reacted with a large excess of MEPB (200 μM) and then diluted for the final measurement, to avoid long incubation times and the inner filter effect. Submicromolar concentrations of H_2_S could be determined with MEPB, even in the presence of an excess of a thiol such as GSH, with improved performance when compared with DNS-Az and HSip-1. Care should be taken to avoid the presence of DTT or other dithiols, which can react with MEPB yielding excimers. In spite of being unsuitable for measuring H_2_S production by enzymes or cells in continuous assays—since the detection should be done with ACN as a cosolvent—MEPB can be used with aliquots taken at fixed times from biochemical samples. The utility of the method is demonstrated with the biochemical systems tested. In the reaction of H_2_S and GSSG that yields GSH and glutathione persulfide, the consumption of H_2_S could be monitored with no interference from thiols. The same was true for the formation of H_2_S by *E. coli* from cysteine (Fig. 7). In addition, the MEPB method can be used with aliquots removed from the headspace of closed reaction containers.

The formation of pyrene excimers from the reaction between H_2_S and MEPB described has several advantages over currently used methods: (i) it can be performed at near-neutral pH, without acidification or alkalinization of the sample, avoiding, for instance, the artifactual release of H_2_S from iron–sulfur clusters; (ii) it does not need specialized equipment; (iii) it is not a laborious procedure since it requires just a simple reaction for 20 min and a dilution; (iv) it is a sensitive method, down to nanomolar levels; (v) it can measure H_2_S even in the presence of thiols, which are a frequent interferent in biochemical systems.

This methodology represents a novel approach to the sensitive and specific detection of H_2_S and provides a valuable tool for analysts in the field of biological chemistry.

## Experimental procedures

### Reagents

Stocks of MEPB (synthesized) were dissolved in HPLC-quality ACN and stored at −20 °C. The concentration was estimated by measuring the absorbance of the pyrene at 345 nm (ε_345_ = 40,000 M^−1^ cm^−1^) (46, 47) of dilutions of the stock in ACN. Stock solutions of H_2_S were prepared by dissolving Na_2_S·9H_2_O salts (Carlo Erba) in water, immediately before use, in sealed vials with minimum headspace. Samples of H_2_S were withdrawn with gas-tight Hamilton syringes. Sealed vials with minimum headspace were used for reactions. GSH, cysteine, sulfite, TCEP, and DTT stock solutions were prepared daily. Dithionite was dissolved in argon-degassed NaOH solutions (0.1 M), and quantified by ferricyanide reduction (49). DHLA was prepared by lipoic acid reduction with excess DTT, separation by solid-phase extraction in a Chromabond C18 cartridge (Macherey–Nagel), washed with 0.1% trifluoroacetic acid in water and then eluted with ACN, and quantified with 5,5′-dithiobis(2-nitrobenzoic acid). Tris buffer 0.1 M, pH 8.5, was used throughout this work unless otherwise is declared. DNS-Az was synthesized according to previous reports (29), and stock solutions were prepared in ethanol. HSip-1 (30) was purchased from Dojindo Molecular Technologies, Inc, and stocks were prepared in distilled water. Its concentration was estimated by measuring the absorbance at 491 nm (ε_491_ = 80,000 M^−1^ cm^−1^) of dilutions of the stock in borate (pH 9.0).

### Synthesis of MEPB

The probe MEPB was synthesized in three steps with good yields as described in section Results—Synthesis of MEPB and Supporting Information. The identity of the products was verified by NMR spectroscopy and high-resolution mass spectrometry (Figs. S2–S6).

### Measurement of H_2_S with MEPB

All reactions and fluorescence measurements involving MEPB were done in Tris–ACN, a 1:1 volume mixture of Tris buffer, 0.1 M, pH 8.5, and ACN, unless indicated otherwise.

The final standard method consisted of reacting H_2_S with 200 μM MEPB in Tris–ACN in a closed vial with minimal headspace for 20 min. The fluorescence measurements were done after diluting the sample 40-fold in Tris–ACN, unless indicated otherwise.

### Fluorescence spectra and measurements

Spectra were recorded in a ChronosFD spectrofluorometer (ISS) equipped with a 300 W high-pressure xenon arc lamp, polarizers, and monochromators, with acquisition at 90°. Both emission spectra (λ_ex_ = 345 nm) or excitation spectra (λ_em_ = 380 or 480 nm, depending on the nature of the fluorophore) were recorded using a slit width of 1 mm (full width at half maximum = 8 nm), unless otherwise stated. Routine measurements were performed in a Varioskan Flash plate reader (Thermo Fisher Scientific) with a xenon flash lamp and monochromators for both excitation and emission.

### Kinetic characterization

Kinetic determinations were performed using either H_2_S or MEPB in excess to study the reactions under pseudo–first-order conditions at 25 °C. As a first approach, solutions of H_2_S (5 μM) in Tris–ACN were incubated with an excess of MEPB (80–300 μM) in sealed vials with minimum headspace. Aliquots were withdrawn at desired incubation times, diluted 1/50 in Tris–ACN, and the spectra were acquired. The reactions were followed during 10 half-lives, and an exponential plus straight-line equation was fitted to the data to obtain the observed rate constants (*k*_obs_). For the sake of clarity, fluorescence intensities divided by the amplitude are presented. Alternatively, using MEPB as the limiting reagent, 500 nM probe in Tris–ACN was mixed with H_2_S (10–985 μM) in a screw cap septum-sealed fluorimeter cuvette, and spectra were recorded every 1 min. The data obtained were analyzed with DynaFit software (42) to estimate the rate constants for the two steps of the reaction (Equations 1 and 2).

### Linearity of the method and limit of detection

The linearity of the method was assessed by treating H_2_S with MEPB in different ranges of concentrations at 25 °C for 20 min in Tris–ACN. Samples were 1/40 diluted and measured in either a plate reader (200 μl in a 96-well plate, bandwidth 5 nm) or a fluorometer (0.2 and 1.0 cm excitation and emission optical pathways, respectively, slit widths of 2 mm). The limits of detection and quantitation were estimated from the slopes obtained in linear regressions of calibration curves and blank measurements as 3*s*_y_/slope and 10*s*_y_/slope, respectively, where *s*_y_ is the standard error of the *y*-intercept or the standard deviation of blanks.

### Evaluation of interferences

GSH, cysteine, sulfite, TCEP, dithionite, H_2_S, DTT, or DHLA (50 μM each) were reacted with MEPB (200 μM) in Tris–ACN for 20 min at 25 °C following the standard method. Emission spectra were recorded to characterize the products.

### Comparison with other fluorescent detection methods

The relative response to H_2_S in the presence of excess GSH was evaluated for three probes: DNS-Az, HSip-1, and MEPB. The measurements were performed according to previous reports or procedures suggested by the supplier (29, 30). H_2_S (20 μM), in the presence of GSH (5–1500 μM), was incubated in phosphate buffer (20 mM, pH 7.5, 0.5% Tween-20) with DNS-Az (200 μM) for 5 min. Then fluorescence intensity at 535 nm (λ_ex_ = 340 nm) was measured in a plate reader. Alternatively, the solutions in PBS were incubated with HSip-1 (120 μM) for 30 min, and the fluorescence intensity was determined at 516 nm (λ_ex_ = 491 nm). The response of MEPB was studied according to the protocol described previously. The intensity of the blank samples (buffer without both H_2_S and GSH) was subtracted from the readings, and the corrected values were normalized to the intensity of the samples of H_2_S in the absence of GSH.

### Monitoring the reaction of H_2_S with GSSG

GSSG (10 mM) was reacted with H_2_S (54 μM) in phosphate buffer (0.1 M, pH 7.4) at 25 °C. Aliquots of 50 μl were withdrawn at different incubation times, reacted with MEPB using the standard method, and measured in a plate reader.

### Monitoring the formation of H_2_S by *E. coli*

*E. coli* BL21 DE3 was grown overnight in LB medium (37 °C, 200 rpm) and diluted to an absorbance of 0.5 at 600 nm in bicine buffer (0.1 M, pH 8.0). Cells were centrifuged (8000*g*, 5 min) and resuspended three times. Cysteine (200 μM) and glucose (2 g/l) were added, and the suspensions were distributed in tubes for incubation (37 °C, 200 rpm). Tubes were centrifuged (20,000*g*, 5 min) at different incubation times, and samples of the supernatant were withdrawn for quantifications. A control without cysteine was run.

## Data availability

All data are contained within the article.

## Supporting information

This article contains supporting information (40, 50, 51, 52, 53, 54, 55, 56, 57, 58, 59).

## Conflict of interest

The authors declare that they have no conflicts of interest with the contents of this article.

## Footnotes

a The term H_2_S is used in this work to refer generically to the mixture of the species H_2_S (hydrogen sulfide) and HS^−^ (hydrosulfide) present in aqueous solution. The concentration of S^2−^ is negligible at working pHs.
