# Supplementary material for: Fluorescent detection of hydrogen sulfide (H2S) through the formation of pyrene excimers enhances H2S quantification in biochemical systems
Source: J Biol Chem. 2022 Aug 19;298(10):102402. doi: 10.1016/j.jbc.2022.102402 (PMC9493391; doi:10.1016/j.jbc.2022.102402)
Supplement: Supporting information [file mmc1.pdf]

SUPPORTING INFORMATION

Fluorescent detection of hydrogen sulfide (H_2S) through the formation of pyrene excimers enhances H_2S quantification in biochemical systems

Manuela Pose[1,2,3], Kearsley M. Dillon[4], Ana Denicola[2,3], Beatriz Alvarez[1,3], John B. Matson[4], Matías N. Möller[2,3,*], Ernesto Cuevasanta[1,3,5,*]

[1]Laboratorio de Enzimología, Instituto de Química Biológica, Facultad de Ciencias, Universidad de la República, Montevideo, Uruguay; [2]Laboratorio de Fisicoquímica Biológica, Instituto de Química Biológica, Facultad de Ciencias, Universidad de la República, Montevideo, Uruguay; [3]Centro de Investigaciones Biomédicas (CEINBIO), Universidad de la República, Montevideo, Uruguay; [4]Department of Chemistry and Macromolecules Innovation Institute, Virginia Tech, Blacksburg, VA 24061, United States; [5]Unidad de Bioquímica Analítica, Centro de Investigaciones Nucleares, Facultad de Ciencias, Universidad de la República, Montevideo, Uruguay

Running title: *Fluorescent detection of H_2S by excimer formation*

[*] To whom correspondence should be addressed: Matías N. Möller: Laboratorio de Fisicoquímica Biológica, Instituto de Química Biológica, Facultad de Ciencias, Universidad de la República, Montevideo, Uruguay, 11400, Tel: (+598) 2525 8618, mmoller@fcien.edu.uy.

[*] To whom correspondence should be addressed: Ernesto Cuevasanta: Laboratorio de Enzimología, Instituto de Química Biológica, Facultad de Ciencias, Universidad de la República, Montevideo, Uruguay, 11400, Tel: (+598) 2525 8618, ecuevasanta@fcien.edu.uy.

Keywords: fluorescence, fluorescent probes, hydrogen sulfide, H_2S, pyrene excimers, quantification

---

Content

Formation of excimers of *N*-(1-pyrene)maleimide by reaction with a dithiol 2
Preparation of MEPB 3
Spectroscopic characterization of the products of MEPB 8
Identification of $(MEPB)_2S$ by HPLC separation 8
Identification of $(MEPB)_2S$ by high-resolution mass spectrometry (HRMS) 9
Solvent effect on excimer formation 10
Evaluation of kinetic models 11
Inner filter effect 12
Evaluation of interferences 13
Monitoring H_2S formation by *E. coli* 14
References 14

Formation of excimers of N-(1-pyrene)maleimide by reaction with a dithiol

Preliminary attempts to detect H_2S were made with the commercially available probe *N*-(1-pyrenyl)maleimide (NPM), which has been previously used for structural characterization of dithiols (1, 2) as well as biophysical characterization of molecular multimers (3–6). When NPM was reacted with the natural thiol glutathione (GSH), the quenching effect of the maleimide was canceled and the fluorescence of the pyrene was significantly enhanced, as expected (7). The fluorescence emission was characterized by two intense peaks at 378 and 398 nm, corresponding to the monomer (Figure S6). When the synthetic dithiol 1,4-dithiothreitol (DTT) was used, the fluorescence emission spectrum was shifted to longer wavelengths, showing a broad peak at 480 nm. This emission is consistent with the dithiol reacting with two molecules of NPM and favoring the formation of excimers (Figure S6) (8, 9). A low emission at 378 and 398 nm could still be observed, probably because of incomplete excimer formation. H_2S also reacted with NPM, but the fluorescence emission was similar to that of GSH (Figure S6), and not to that of DTT, indicating that no excimers were formed. H_2S is expected to react with a molecule of NPM to form an intermediary thiol that would then react with a second molecule of NPM, yielding a thioether-bridged NPM dimer. The absence of excimer fluorescence suggested that the two pyrenes covalently bound to the sulfur suffered steric hindrance that prevented the formation of excimers and that a longer linker region was necessary to successfully form excimers from the reaction with H_2S. Thus, we decided to synthesize a probe that contained a maleimide group and a pyrene linked by a medium-length chain: MEPB.

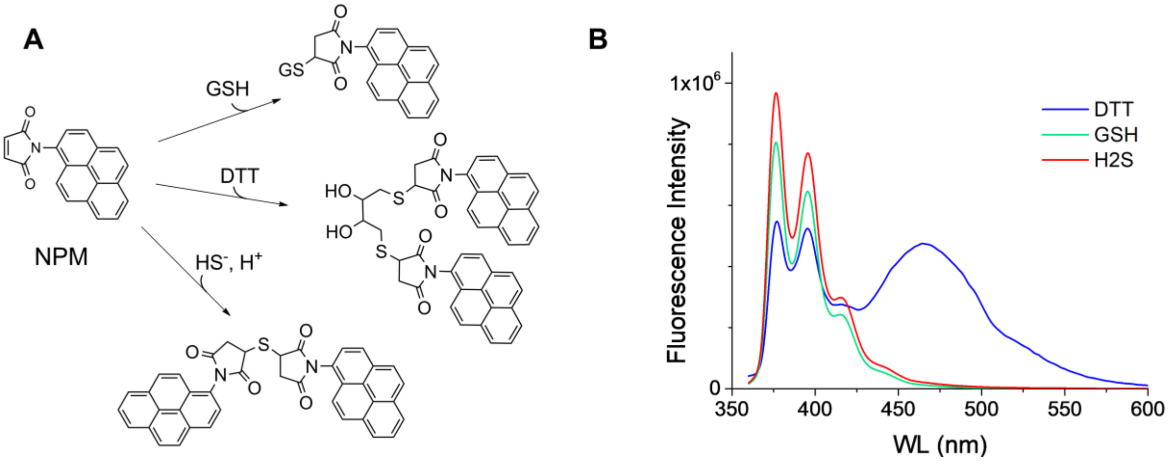

Figure S1: Excimer formation with NPM. A) Proposed reaction for monothiols, dithiols and H_2S with NPM. NPM reacts with the monothiol GSH to form the monoalkyl derivative. Dithiols like DTT react with two molecules of NPM to form a bridged dimer. H_2S reacts with one molecule of NPM, forming an intermediate thiol that then reacts with a second NPM to form the thioether-bridged dimer. **B)** Emission spectra (λ_{ex} 345 nm, technical, no polarizers) of mixtures of NPM (200 μM) with H_2S, GSH, or DTT (50 μM), incubated for 20 min at 25 °C in Tris-ACN (1:1 volume mixture of Tris buffer -0.1 M, pH 8.5- and acetonitrile), and diluted 1/400 before measurement in the ISS instrument.

Preparation of MEPB

The probe MEPB was synthesized in three steps with good yields. The identity of the products was verified by NMR spectroscopy and HRMS (Figures S2-6).

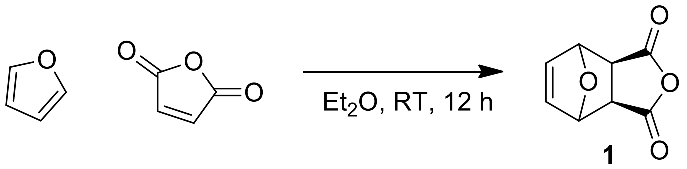

Synthesis of *exo* oxanorbornene anhydride (1). To a 250 ml, flame-dried, two-neck round bottom flask equipped with a septum and an N_2 inlet, maleic anhydride (10.8 g, 110 mmol), and Et_2O (70 ml) were added under N_2 flow. Furan (22 ml, 311 mmol) was then added directly to the flask from a graduated cylinder. The flask was sealed with a septum, and the reaction mixture was allowed to stir for 12 h at RT under N_2 flow. A white precipitate formed that was collected by filtration and washed with 50 ml cold Et_2O. This white powder (16.1 g, 88% yield) was used in the next step without further purification. NMR spectra were consistent with previous reports (10).

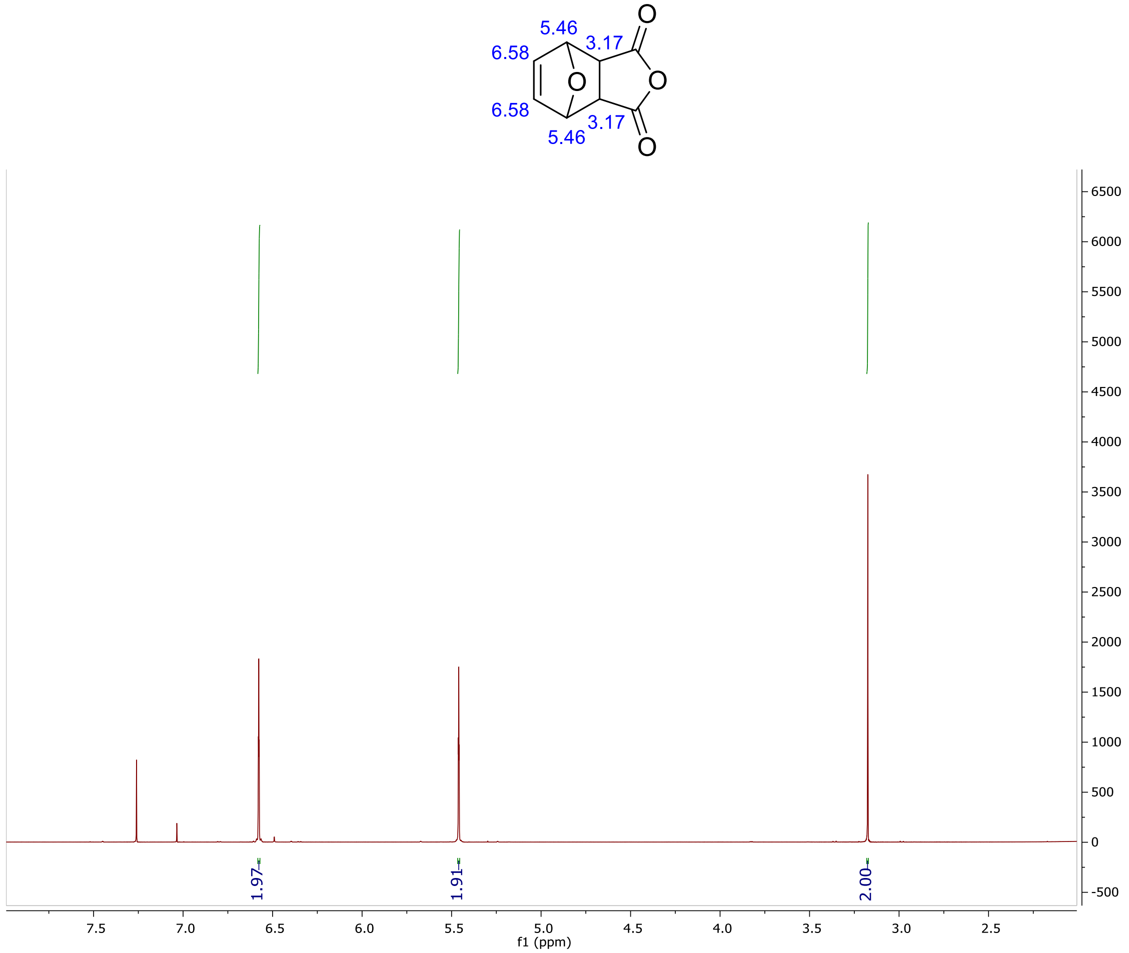

Figure S2: ^{1}H-NMR spectrum of compound (1) (δ, $CDCl_3$). 6.58 (2H, t, J = 1.0 Hz), 5.46 (2H, t, J = 1.0 Hz), 3.17 (2H, s).

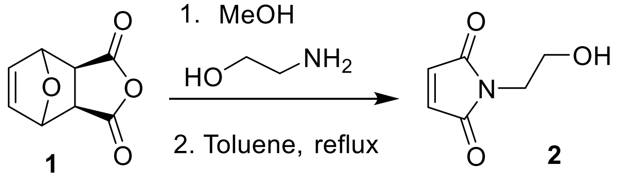

Synthesis of *N*-(2-hydroxyethyl)maleimide (2). To a flame-dried round bottom flask, anhydride (**1**) (8.7 g, 52 mmol) in degassed methanol (80 ml) was transferred via cannula from a Strauss flask, and the solution was cooled to 0 °C on an ice bath. A solution of ethanolamine (3.5 ml, 58 mmol) in degassed methanol (15 ml) was dripped in slowly with an addition funnel over 30 min. The reaction mixture was allowed to warm up to RT and stirred for 1 h. A reflux condenser was then added to the flask, and the reaction mixture was heated at reflux for 16 h. The reaction mixture was then concentrated via a rotary evaporator to yield an off-white solid, which was taken up in CH_2Cl_2 (100 ml) and washed with water in a separatory funnel (3 x 25 ml). The organic layer was removed, dried over Na_2SO_4, and concentrated in vacuo to yield a white powder, which was immediately carried to the next reaction step without further purification. Next, degassed toluene (80 ml) and a reflux condenser were added to the 250 ml round bottom flask containing the first product, and the reaction mixture was heated at reflux for 16 h under positive nitrogen flow. A distillation apparatus was assembled, and distillation was carried out at 90 °C for 8 h at atmospheric pressure to remove excess furan and prevent Diels-Alder recombination. Once furan had ceased distilling, the remaining toluene was removed via rotary evaporation to yield the desired product as off-white crystals (6.3 g, 79% yield). NMR spectra were consistent with previous reports (11).

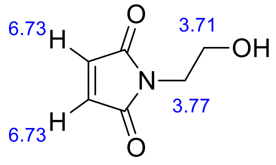

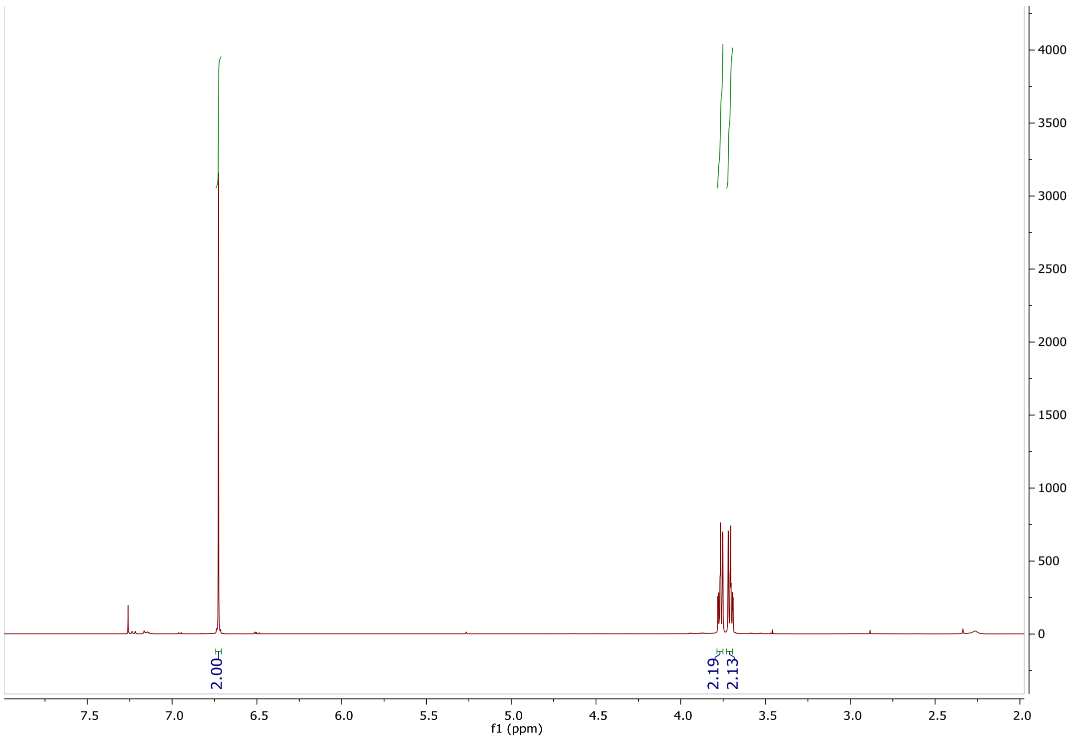

Figure S3: ^{1}H-NMR spectrum of compound (2) (δ, $CDCl_3$). 6.73 (2H, s), 3.77 (2H, m), 3.71 (2H, m).

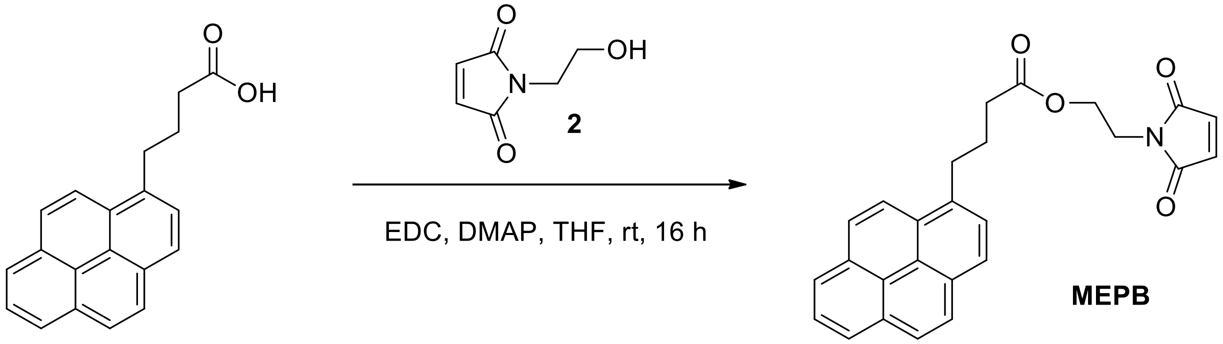

Synthesis of 2-(maleimido)ethyl 4-(pyren-1-yl)butanoate (MEPB). To a flame-dried, two-neck, round bottom flask, equipped with a stopper and a septum, 4-(1-pyrenyl)butyric acid (150 mg, 0.52 mmol) and anhydrous THF (10 ml, via cannula from a Strauss flask) were added under N_2 flow. To this suspension, ethyl-3-(3-dimethylaminopropyl)carbodiimide (EDC, 400 mg, 2.5 mmol) and 4-dimethylaminopyridine (DMAP, 3.0 mg, 26 µmol) were added. The reaction mixture was allowed to stir for 30 min at RT until homogeneous. To this amber solution, *N*-(2-hydroxyethyl)maleimide **(2)** (77 mg, 0.56 mmol) was added under N_2 flow, and the reaction mixture was allowed to stir for 16 h at RT, monitoring reaction progress by TLC (DCM, UV visualization). The reaction mixture was then concentrated via rotary evaporation to yield an orange waxy solid, which was taken up in CH_2Cl_2 (20 ml) and then washed with 1 N HCl (10 ml), deionized water (2 x 10 ml), and brine (10 ml). The organic layer was then removed and dried over Na_2SO_4. The product was dry-loaded onto silica and purified by column chromatography, eluting with 25% EtOAc in hexanes (Spot 2, R_f = 0.3, visualized with UV) to yield the product as a yellow waxy solid (140 mg, 65% yield).

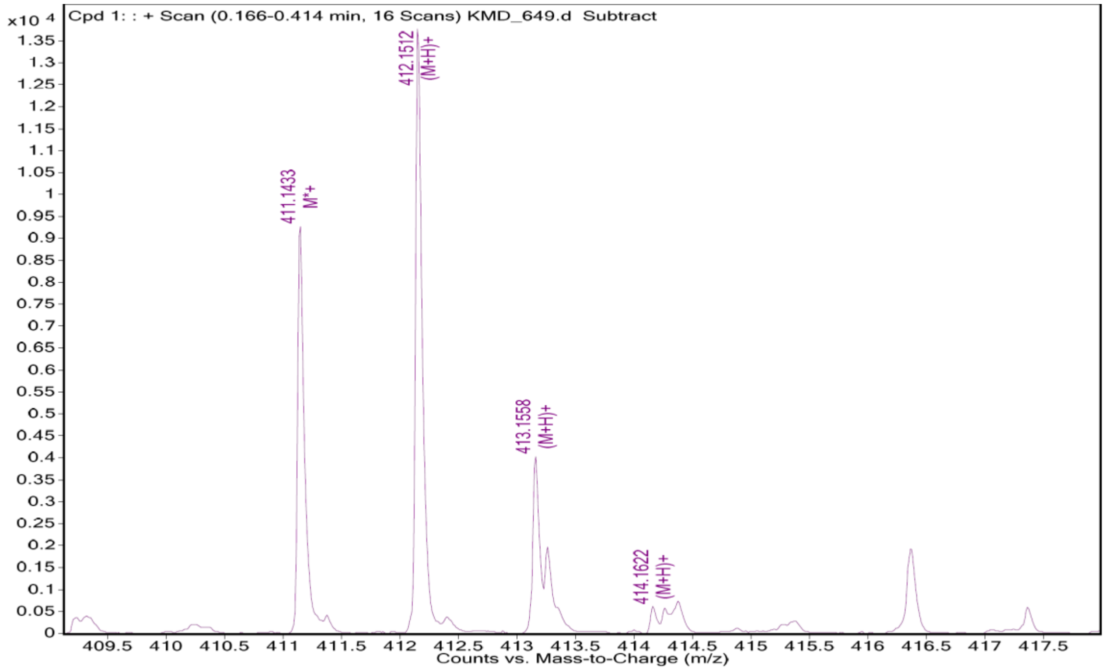

Figure S4: HRMS (ESI-TOF). Calculated for $C_{26}H_{21}NO_4$ $[M+H]^+$ 412.1548, found 412.1512.

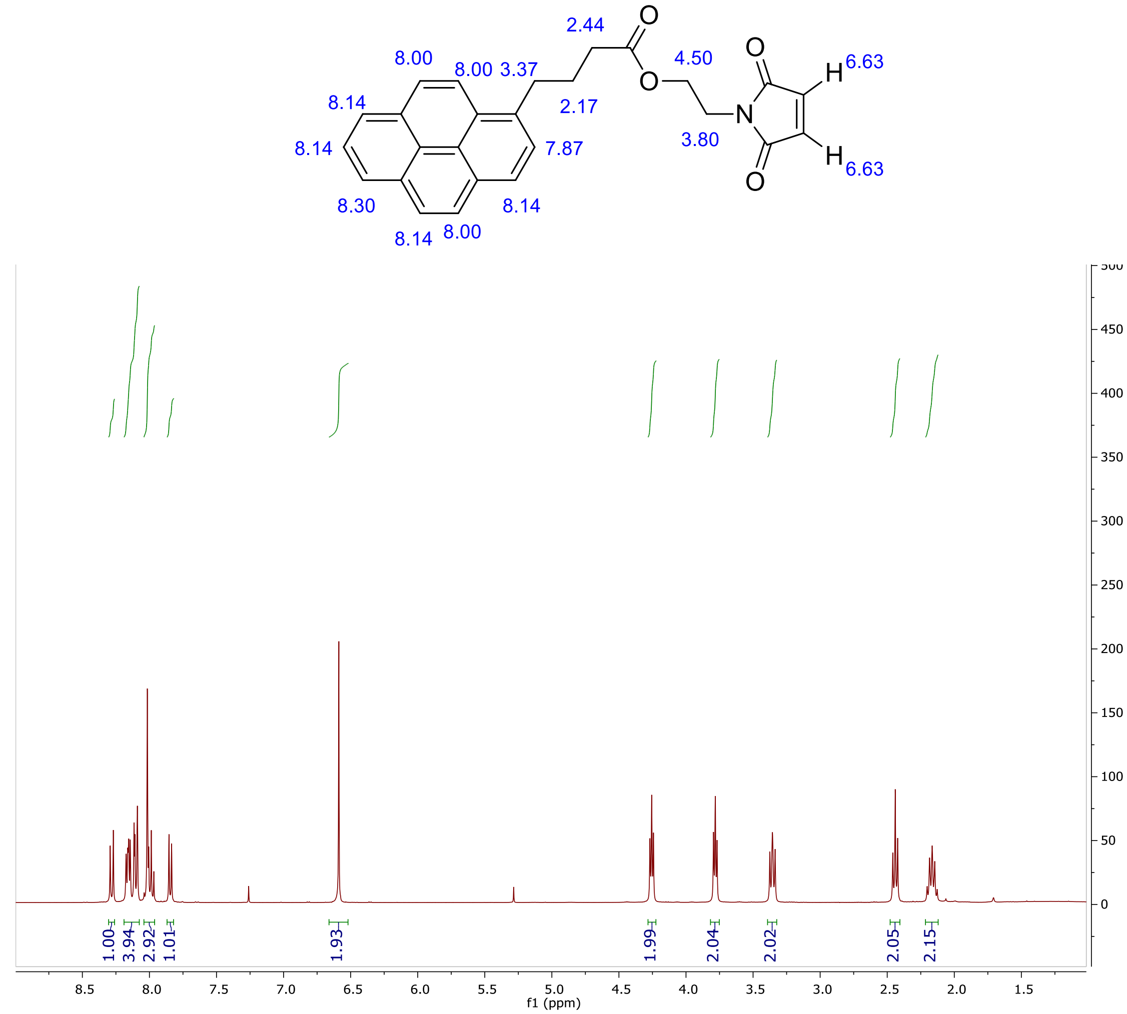

Figure S5: ^{1}H-NMR spectrum of compound MEPB (δ, $CDCl_3$). 8.30 (1H, d, *J* = 9.5 Hz), 8.18-8.10 (4H, m), 8.02-7.97 (3H, m), 7.87 (1H, d, *J* = 7.7 Hz), 6.63 (2H, s), 4.5 (2H, t, *J* = 5.3 Hz) 3.8 (2H, t, *J* =5.3 Hz), 3.37 (2H, t, *J* = 7.6 Hz), 2.44 (2H, t, *J* = 7.5 Hz), 2.17 (2H, quint, *J* = 7.6 Hz).

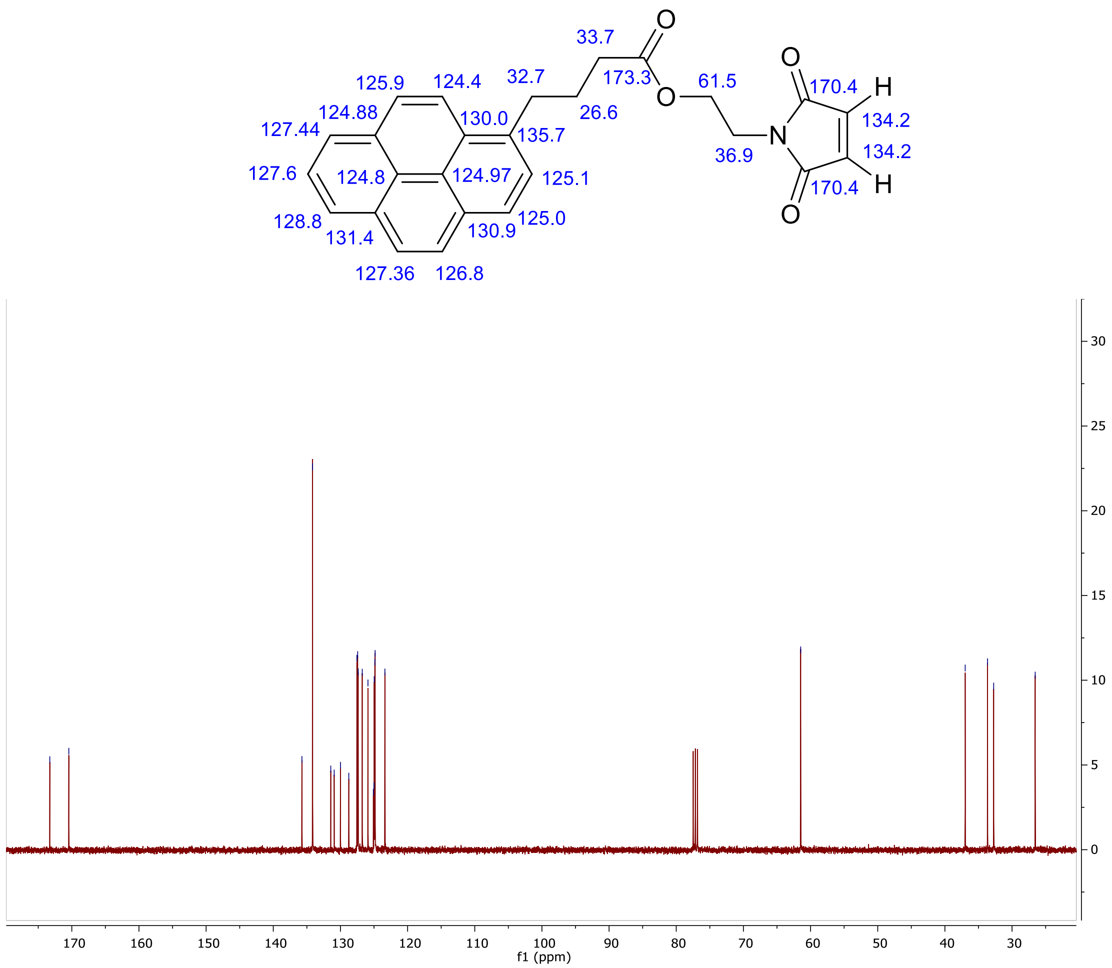

Figure S6: ^{13}C-NMR spectrum of compound MEPB (δ, $CDCl_3$). 173.3, 170.4, 135.7, 134.2, 131.4, 130.9, 130.0, 128.8, 127.6, 127.44, 127.36, 126.8, 125.9, 125.1, 125.0, 124.97, 124.88, 124.83, 124.4, 61.5, 36.9, 33.7, 32.7, 26.6.

Spectroscopic characterization of the products of MEPB

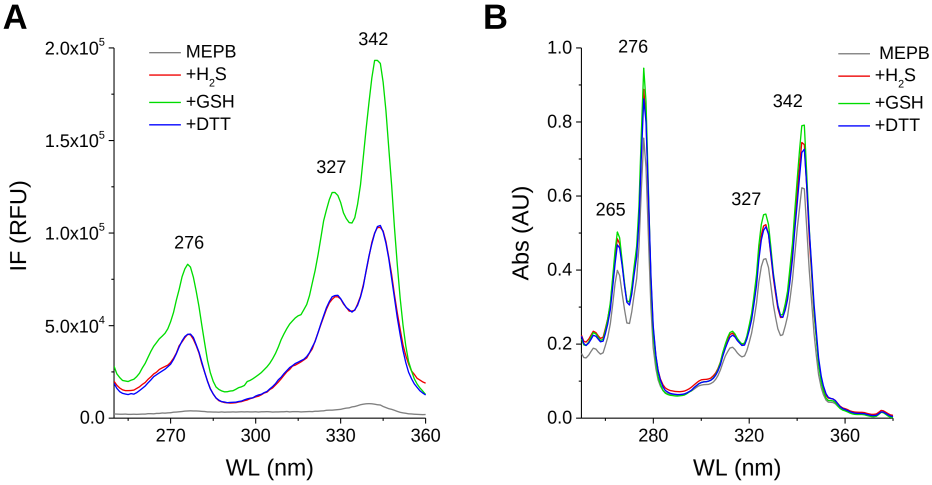

Figure S7: Spectroscopic characterization of the products. A) Excitation spectra of the products of the reactions with MEPB. Spectra obtained for MEPB alone (25 µM) and after reacting with GSH (25 µM, λ_{em} 380 nm), H_2S, or DTT (12,5 µM, λ_{em} 480 nm). The mixtures in Tris-ACN were incubated for 20 min at 25 °C and diluted 1/400. **B)** Absorption spectra of the products of the reactions with MEPB. Spectra obtained for MEPB alone (25 µM) and after reacting with GSH (25 µM), H_2S, or DTT (12,5 µM) in Tris-ACN incubated for 20 min at 25 °C.

Identification of $(MEPB)_2S$ by HPLC separation

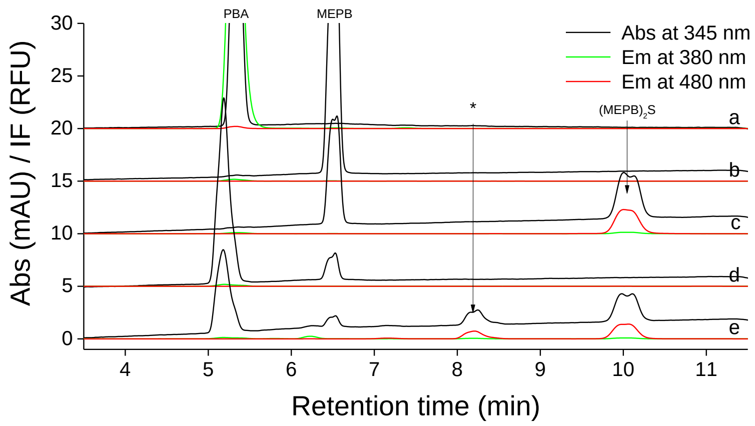

Figure S8: Separation of $(MEPB)_2S$ by reversed-phase liquid chromatography. Representative chromatograms obtained by either absorbance detection at 345 nm or fluorescence emission recording at 380 and 480 nm (λ_{ex} 345 nm). Analysis was performed in a 1260 Infinity HPLC (Agilent Technologies), equipped with a C18 Ascentis column (100 × 4.6 mm, 3 µm, Sigma-Aldrich), a diode array absorbance detector (1260 DAD VL, Agilent), and a fluorescence detector (1260 FLD, Agilent). Controls included direct injection of 5 µl of **(a)** 4-(1-pyrenyl)butyric acid (PBA, 20 µM) or **(b)** MEPB (20 µM) in Tris-ACN. **(c)** MEPB (200 µM) was mixed with H_2S (50 µM) for 20 min at 25 °C, diluted 1/10 in Tris-ACN, and aliquots of 5 µl were injected. Alternatively, MEPB or the mixture containing the $(MEPB)_2S$ were diluted in carbonate buffer (0.1 M, pH 11.0)/ACN (1:1) for 10 min (**d** and **e**, respectively). Hydrolysis products are evident after incubation at pH 11, but did not match PBA and

were not further studied. The HPLC was set at a flow rate of 0.8 ml/min with a mobile phase consisting of 0.1% trifluoroacetic acid in water and ACN (40:60 from 0 to 0.5 min, changing to 5:95 from 4 to 9 min, 40:60 from 10 to 14 min).

Identification of $(MEPB)_2S$ by high-resolution mass spectrometry (HRMS)

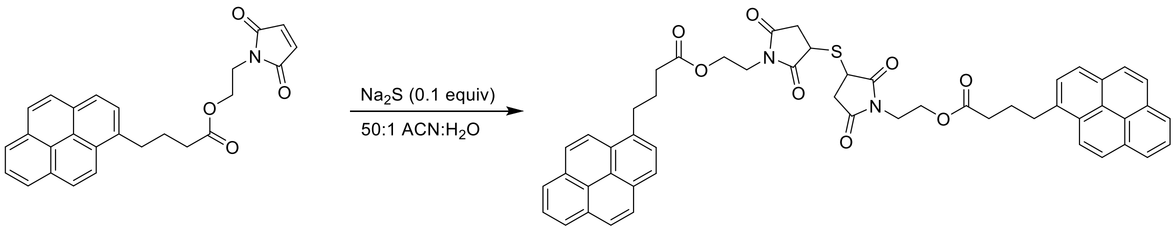

MEPB (1.0 mg, 2.4 µmol) was dissolved in LC-MS grade ACN (1.0 ml), creating a clear, colorless solution. Na_2S (1.0 mg), stored in a glove box under a nitrogen atmosphere, was dissolved in LC-MS grade H_2O (1.0 ml). 20 µl Na_2S stock solution (0.26 µmol) was then injected into the solution of MEPB. The resulting pink solution was analyzed by HRMS.

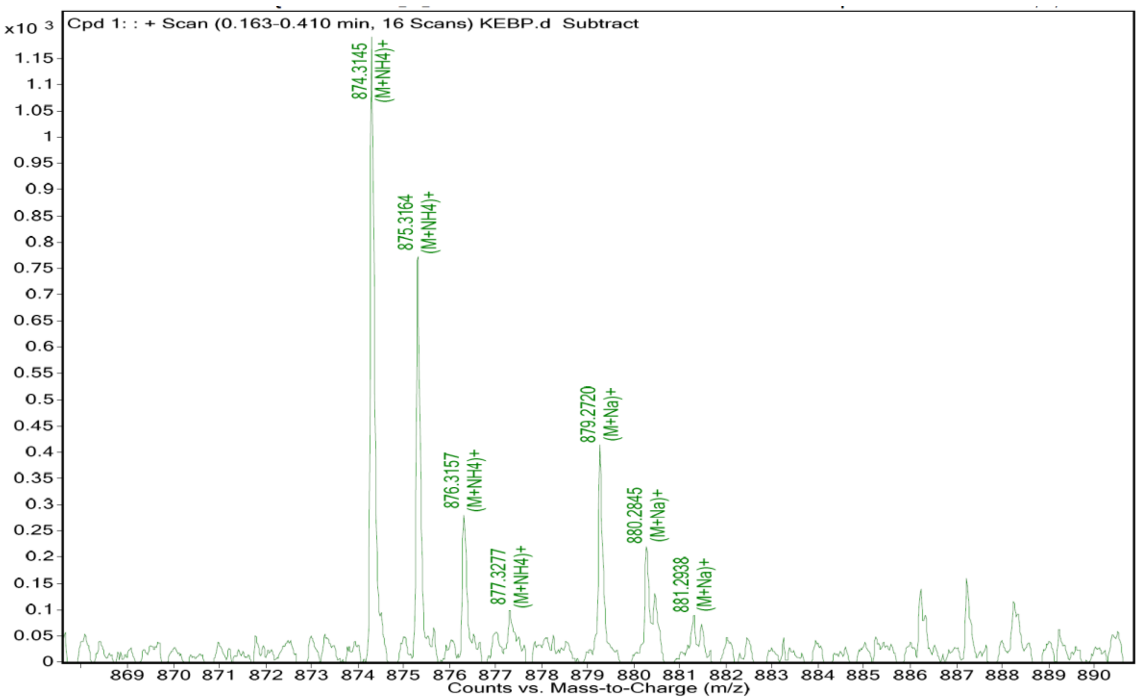

Figure S9: MS (ESI-TOF) spectrum of $(MEPB)_2S$. A peak was observed at m/z = 874.3145, consistent with the $[M+NH_4]^+$ adduct of the expected product (calculated m/z = 874.3162). The other peak observed at m/z = 879.2720 was consistent with the sodium adduct $[M+Na]^+$ (calculated 879.2716).

Solvent effect on excimer formation

The effect of the solvent on the fluorescence emission was studied. When dilutions were done in pure ACN, the GS-MEPB product formed excimers even without covalent dimer linkage, likely because the low solubility of the tripeptide in this solvent favored the spontaneous aggregation. In contrast, in pure ACN the $(MEPB)_2S$ product formed less excimer, likely because the hydrophobic effect driving the formation of excimer was low (Figure S10). When dilutions were done in Tris buffer alone, no excimer emission was observed for H_2S- or DTT-MEPB products, probably because the solubility of the pyrenes is low and covalent dimers precipitated (Figure S10). The 1:1 mixture of ACN and Tris buffer balanced these effects and allowed the differentiation of the products, modulating the solubility of the components and avoiding the non-specific formation of excimers.

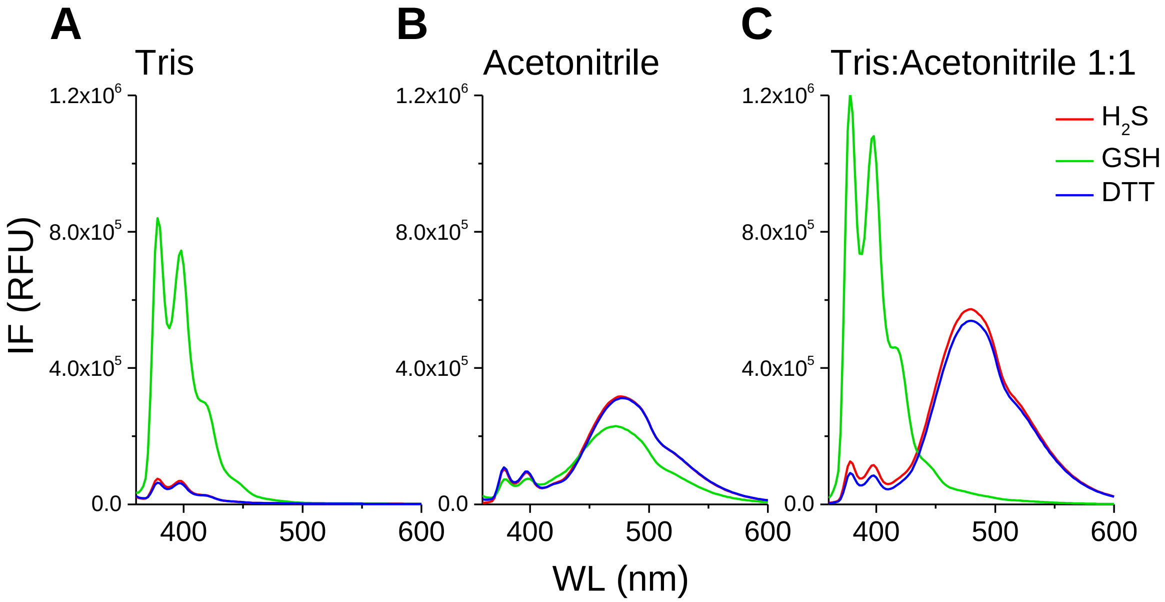

Figure S10: Effect of the composition of the solvent on the emission of the excimers. Emission spectra (λ_{ex} 345 nm) of the reaction mixtures containing MEPB (200 µM) with either H_2S (50 µM, black), GSH (50 µM, red) or DTT (50 µM, blue) in Tris-ACN for 20 min at 25 °C. Then, aliquots were diluted (1/50) using different solvent proportions: **A)** a highly polar solvent (Tris buffer (0,1 M, pH 8,5)), **B)** a solvent of lower polarity (ACN) and **C)** the mixture Tris (pH 8,5)/ACN (1:1).

Evaluation of kinetic models

The reaction models assessed included reversible and irreversible steps for the reactions of H_2S and the intermediate thiol with MEPB. Other reactions that were considered were the hydrolysis of the probe MEPB and of $(MEPB)_2S$.

Model 1:

$MEPB + H_2S \rightarrow MEPB\text{-}SH$	k_1
$MEPB\text{-}SH + MEPB \rightarrow (MEPB)_2S$	k_2

Model 2:

$MEPB + H_2S \rightarrow MEPB\text{-}SH$	k_1
$MEPB\text{-}SH + MEPB \rightleftharpoons (MEPB)_2S$	k_{-2}, k_2

Model 3:

$MEPB + H_2S \rightleftharpoons MEPB\text{-}SH$	k_{-1}, k_1
$MEPB\text{-}SH + MEPB \rightarrow (MEPB)_2S$	k_2

Model 4:

$MEPB + H_2S \rightleftharpoons MEPB\text{-}SH$	k_{-1}, k_1
$MEPB\text{-}SH + MEPB \rightleftharpoons (MEPB)_2S$	k_{-2}, k_2

Model 5:

$MEPB + H_2S \rightarrow MEPB\text{-}SH$	k_1
$MEPB\text{-}SH + MEPB \rightarrow (MEPB)_2S$	k_2
$MEPB \rightarrow hydrolysis\ products$	k_3

Model 6:

$MEPB + H_2S \rightleftharpoons MEPB\text{-}SH$	k_{-1}, k_1
$MEPB\text{-}SH + MEPB \rightleftharpoons (MEPB)_2S$	k_{-2}, k_2
$MEPB \rightarrow hydrolysis\ products$	k_3

Model 7:

$MEPB + H_2S \rightarrow MEPB\text{-}SH$	k_1
$MEPB\text{-}SH + MEPB \rightarrow (MEPB)_2S$	k_2
$MEPB \rightarrow hydrolysis\ products$	k_3
$(MEPB)_2\text{-}S \rightarrow hydrolysis\ products$	k_4

Model 8:

$MEPB + H_2S \rightleftharpoons MEPB\text{-}SH$	k_{-1}, k_1
$MEPB\text{-}SH + MEPB \rightleftharpoons (MEPB)_2S$	k_{-2}, k_2
$MEPB \rightarrow hydrolysis\ products$	k_3
$(MEPB)_2\text{-}S \rightarrow hydrolysis\ products$	k_4

Datasets of fluorescence *vs.* time were studied by using the model discrimination analysis of Dynafit (BioKin, Ltd.). The initial concentrations of MEPB and H_2S were considered, and the rate constants were set as variables. Fluorescence contribution at 480 nm was attributed only to $(MEPB)_2S$.

According to the report based on the Akaike information criterion (AIC), model 4 was chosen.

Model number	Number of parameters	Delta AIC
4	4	0
7	3	33.6
5	3	147.9
2	3	188.5
3	3	428.4
1	2	448

Alkaline hydrolysis

The stability of MEPB at alkaline pH was tested to find the optimal pH for H_2S assays. At higher pH H_2S will be ionized and, therefore, volatility will be prevented, facilitating the analysis of the samples, but the maleimide in MEPB may hydrolyze, or less likely the ester bond. It was found that MEPB was stable against hydrolysis up to pH 8.5 for up to 2 hours (Figure S11).

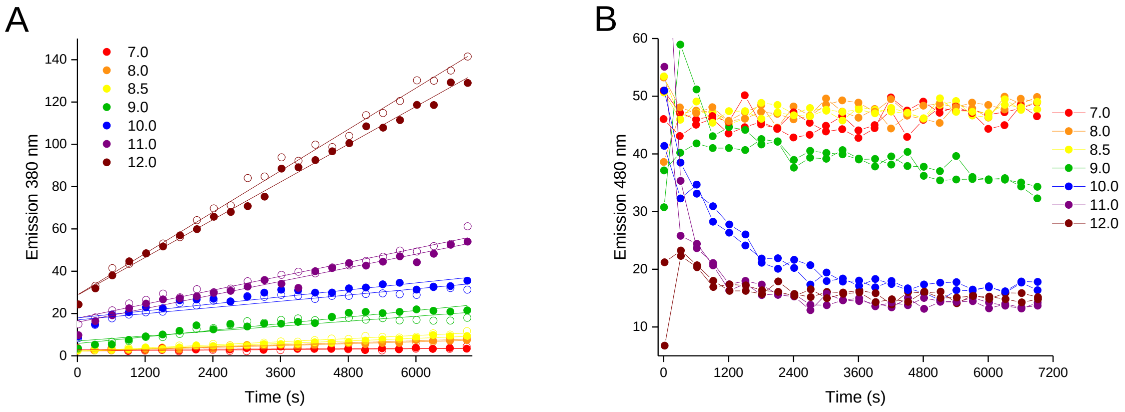

Figure S11: Effect of basicity on the stability of the probe. A) Fluorescence emission of MEPB (5 µM), or **B)** $(MEPB)_2S$ (0. 5 µM) and MEPB (4 µM) (previously synthesized in Tris-ACN), during incubation in different buffer/ACN (1:1) dilutions. Different pHs were kept constant with phosphate (0.1 M, pH 7.0), Tris (0.1 M, pHs 8.0 and 8.5), borate (0.1 M, pHs 9.0 and 10.0), and carbonate buffer (0.1 M, pHs 11.0 and 12.0). The excitation wavelength was set at 345 nm and emission at 380 and 480 nm was acquired in a plate reader.

Inner filter effect

To control for the inner filter effect, the emission of the pre-synthesized dimer ($(MEPB)_2S$) was studied at different concentrations. $(MEPB)_2S$ was obtained by incubating H_2S (10 µM) with MEPB (200 µM). Serial dilutions of the mixture were prepared to decrease the pyrene concentration using Tris-ACN and the fluorescence emission was recorded at 480 nm.

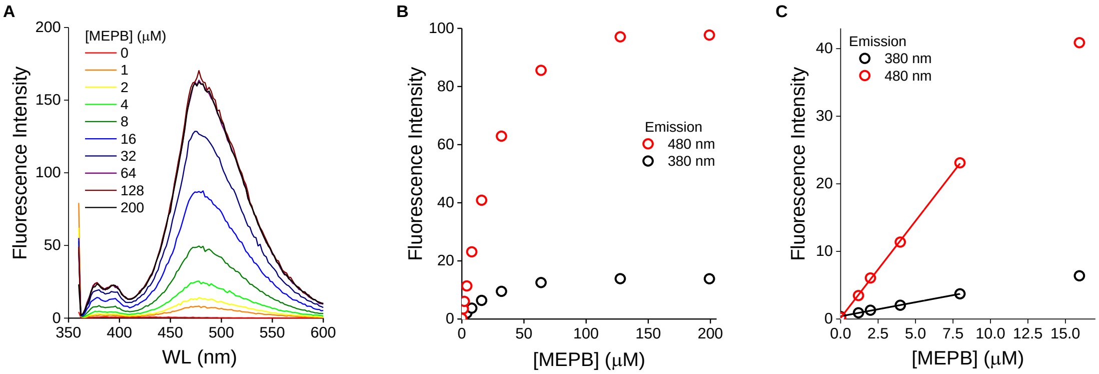

Figure S12: Inner filter effect. A stock mixture of MEPB (200 µM) with H_2S (10 µM) in HEPES (0.1 M, pH 7.4)/ACN (1:1) was incubated at RT for 15 min and diluted with Tris-ACN. **A)** Emission spectra of dilutions. The excitation wavelength was set at 342 nm and spectra were acquired in a plate reader (Varioskan) using 96-well black plates (250 µl). **B)** Dependence of fluorescence intensity of excimers on final pyrene concentration. **C)** Linear range where the inner filter effect is not observed, up to 8 µM MEPB, final concentration.

Evaluation of interferences

GSH, cysteine, thiosulfate, DTT, H_2S, or TCEP (50 µM each) were reacted with MEPB (200 µM) in Tris-ACN for 20 min at 25 °C. Samples were diluted (1/400 for ChronosFD or 1/40 for Varioskan Flash) with the same solvent and emission spectra were recorded to characterize the products. Moreover, H_2S (5 µM) was incubated with GSH (0.5-50 µM) and MEPB (200 µM), or, alternatively, GSH (50 µM) was incubated with H_2S (1.6-20 µM), under the same conditions.

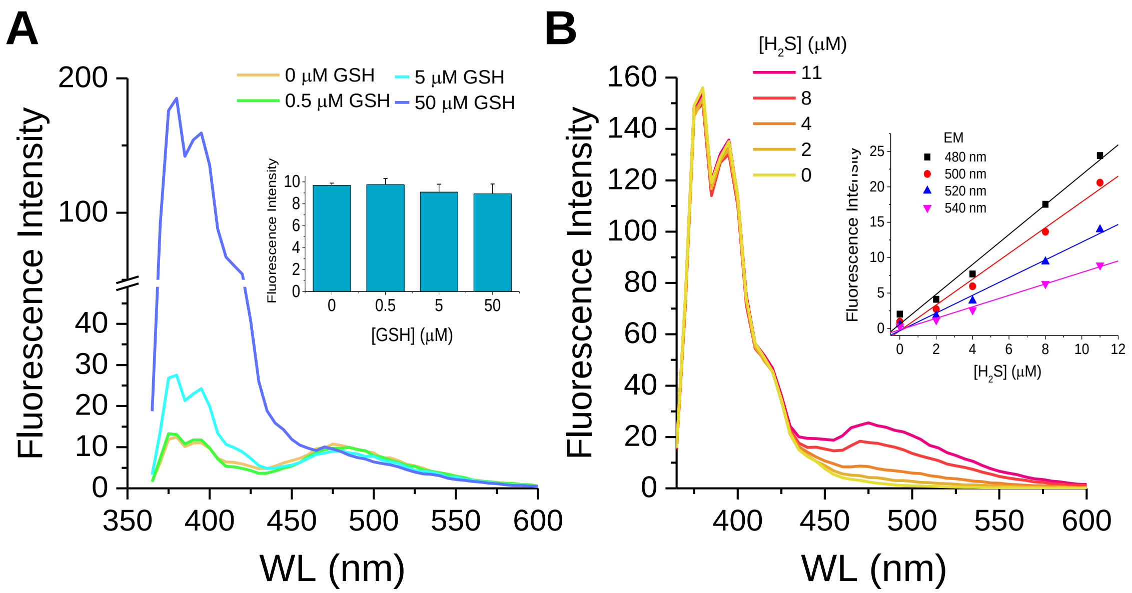

Figure S13: Comparison of the emission spectra of MEPB with thiols. A) Interference produced by thiols. Emission (λ_{ex} 345 nm) of mixtures of MEPB (200 µM), H_2S (5 µM) and increasing concentrations of GSH (0-50 µM) in Tris-ACN, incubated for 20 min at 25 °C and diluted 1/40. The Inset shows that the emission of excimers is not affected by 10-fold excess GSH (n = 3, average ± standard deviation). **B)** Emission spectra (λ_{ex} 345 nm) of MEPB reaction with GSH and H_2S. The Inset shows the calibration curve for H_2S (0-11 µM) performed in the presence of GSH (50 µM) at different wavelengths (representative result, n = 3).

Monitoring H_2S formation by E. coli

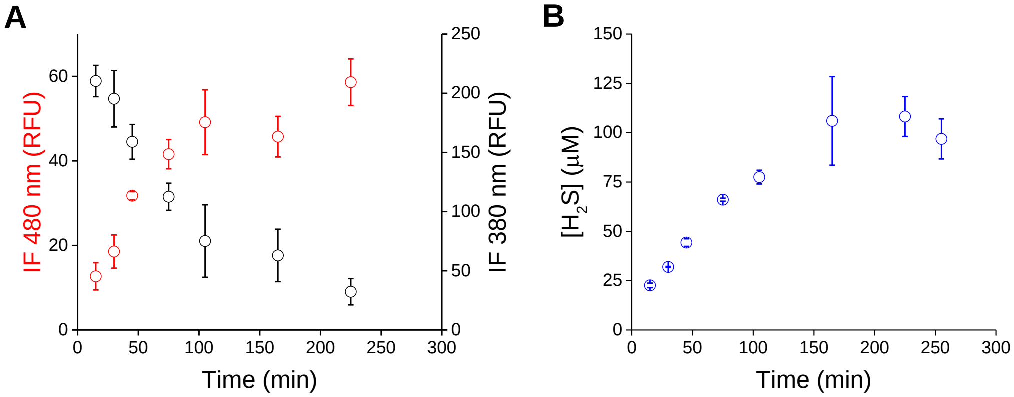

Figure S15: Monitoring H_2S formation by *E. coli*. A) Fluorescence emission at 480 nm (red) and 380 nm (black) of supernatants treated with MEPB to obtain data for Figure 6B (λ_{ex} 345 nm, n = 3, average ± standard deviation). Emission at 380 nm (monomer) approximates the level of cysteine remaining in the solution. **B)** Analysis of H_2S with the methylene method in supernatants of *E. coli* supplemented with cysteine and glucose (experimental conditions identical to Figure 6B). Aliquots were mixed with *N*,*N*-dimethyl-*p*-phenylenediamine and ferric chloride in HCl (3 mM, 4.5 mM and 1.25 M, respectively, final concentrations). The absorbance at 670 nm was determined after 20 min incubations at room temperature. Calibration curves with $Na_2S \cdot 9H_2O$ were performed.

References

1. Yoshida, H., Sudo, M., Todoroki, K., Nohta, H., and Yamaguchi, M. (2009) Highly selective and simple method for determination of polythiols based on liquid chromatography with postcolumn excimer fluorescence derivatization. *Analytical Sciences*. **25**, 829–832
2. Niwayama, S., Kassar, A. S., Zhao, T., Sutton, R. B., and Altenberg, G. A. (2011) A pyrene maleimide with a flexible linker for sampling of longer inter-thiol distances by excimer formation. *PLOS ONE*. **6**, e26691
3. West, J. M., Tsuruta, H., and Kantrowitz, E. R. (2004) A fluorescent probe-labeled Escherichia coli aspartate transcarbamoylase that monitors the allosteric conformational state. *J. Biol. Chem*. **279**, 945–951
4. Patel, A. B., Khumsupan, P., and Narayanaswami, V. (2010) Pyrene fluorescence analysis offers new insights into the conformation of the lipoprotein-binding domain of human apolipoprotein E. *Biochemistry*. **49**, 1766–1775
5. Mizuguchi, C., Hata, M., Dhanasekaran, P., Nickel, M., Phillips, M. C., Lund-Katz, S., and Saito, H. (2012) Fluorescence analysis of the lipid binding-induced conformational change of apolipoprotein E4. *Biochemistry*. **51**, 5580–5588
6. Bains, G. K., Kim, S. H., Sorin, E. J., and Narayanaswami, V. (2012) The extent of pyrene excimer fluorescence emission is a reflector of distance and flexibility: analysis of the segment linking the LDL receptor-binding and tetramerization domains of apolipoprotein E3. *Biochemistry*. **51**, 6207–6219
7. Jameson, D. M. (2014) *Introduction to Fluorescence*, CRC Press, 10.1201/b16502
8. Lehrer, S. S. (1995) Pyrene excimer fluorescence as a probe of protein conformational change. *Subcell Biochem*. **24**, 115–132
9. Bains, G., Patel, A. B., and Narayanaswami, V. (2011) Pyrene: a probe to study protein conformation and conformational changes. *Molecules*. **16**, 7909–7935

10. McCluskey, A., Ackland, S. P., Bowyer, M. C., Baldwin, M. L., Garner, J., Walkom, C. C., and Sakoff, J. A. (2003) Cantharidin analogues: synthesis and evaluation of growth inhibition in a panel of selected tumour cell lines. *Bioorganic Chemistry*. **31**, 68–79
11. Linares, I. A. P., de Oliveira, K. T., and Perussi, J. R. (2017) Chlorin derivatives sterically-prevented from self-aggregation with high antitumor activity for photodynamic therapy. *Dyes and Pigments*. **145**, 518–527
